# 3D In Vitro Blood‐Brain‐Barrier Model for Investigating Barrier Insults

**DOI:** 10.1002/advs.202205752

**Published:** 2023-02-13

**Authors:** Wei Wei, Fernando Cardes, Andreas Hierlemann, Mario M. Modena

**Affiliations:** ^1^ ETH Zürich Department of Biosystems Science and Engineering Bio Engineering Laboratory Basel 4058 Switzerland

**Keywords:** 3D models, blood‐brain‐barrier, cerebral ischemia, microfluidics, transendothelial electrical resistance (TEER)

## Abstract

Blood‐brain‐barrier (BBB) disruption has been associated with a variety of central‐nervous‐system diseases. In vitro BBB models enable to investigate how the barrier reacts to external injury events, commonly referred to as insults. Here, a human‐cell‐based BBB platform with integrated, transparent electrodes to monitor barrier tightness in real time at high resolution is presented. The BBB model includes human cerebral endothelial cells and primary pericytes and astrocytes in a 3D arrangement within a pump‐free, open‐microfluidic platform. With this platform, this study demonstrates that oxygen‐glucose deprivation (OGD), which mimics the characteristics of an ischemic insult, induces a rapid remodeling of the cellular actin structures and subsequent morphological changes in the endothelial cells. High‐resolution live imaging shows the formation of large actin stress‐fiber bundles in the endothelial layer during OGD application, which ultimately leads to cell shrinkage and barrier breakage. Simultaneous electrical measurements evidence a rapid decrease of the barrier electrical resistance before the appearance of stress fibers, which indicates that the barrier function is compromised already before the appearance of drastic morphological changes. The results demonstrate that the BBB platform recapitulates the main barrier functions in vitro and can be used to investigate rapid reorganization of the BBB upon application of external stimuli.

## Graphical abstract

A human‐cell‐based, open‐microfluidic blood‐brain‐barrier platform with integrated, transparent electrodes to monitor barrier tightness in real time via impedance and high‐resolution imaging. The platform is used to investigate how the barrier reacts to external injury events, such as ischemic insults. Electrical measurements show a rapid decrease of the barrier electrical resistance before the appearance of actin stress‐fiber bundles during the ischemic insult.

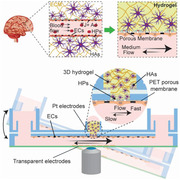

## 1 Introduction

The blood–brain barrier (BBB) is a specialized system of brain microvasculature that tightly regulates the transfer of nutrients, metabolites, and ions between the blood circulation system and the central nervous system (CNS) to maintain brain homeostasis.^[^ ^1^, ^2^, ^3^, ^4^ ^]^ The high selectivity of the barrier protects the brain from potentially toxic substances and pathogens in the blood, and enables the removal of waste products from the brain, thereby acting as the first‐line protection of the CNS. Insults to the BBB including physical injuries, such as strokes and traumatic brain injuries, drastically affect the function and tightness of the BBB, which, in turn, has direct effects on the CNS.^[^ ^5^, ^6^, ^7^, ^8^ ^]^ Furthermore, the high selectivity of the BBB also represents a major roadblock in the effective delivery of drugs and therapeutics to treat CNS diseases,^[^ ^9^, ^10^, ^11^ ^]^ including neurodegenerative diseases, cerebrovascular diseases, and brain tumors.^[^ ^4^, ^12^ ^]^

To improve the understanding of the BBB transport mechanisms and its response to external stress events, various static and dynamic in vitro BBB models have been reported in literature.^[^ ^13^, ^14^, ^15^, ^16^, ^17^, ^18^, ^19^, ^20^, ^21^, ^22^ ^]^ These models aim at recapitulating morphological and biochemical characteristics of the BBB under controllable and reproducible conditions.^[^ ^2^, ^23^ ^]^ Compared to using in vivo animal models, in vitro models offer lower costs, higher throughput, and the possibility to use human‐based cellular models, which reduces potential issues related to specie‐dependent differences between human and animal BBBs. In addition, in vitro models have been instrumental in reducing animal use to address ethical concerns.^[^ ^24^, ^25^, ^26^ ^]^

The most commonly used in vitro BBB models rely on transwell systems, which include the static culturing of an endothelial‐cell barrier on a porous membrane that is immersed in a medium within a well.^[^ ^13^, ^14^, ^16^ ^]^ The simple parallelization of the culture system and its compatibility with a well‐plate format have greatly promoted the use of this approach. However, static culturing in a well plate cannot recapitulate important morphological and physiological aspects of the BBB environment, such as liquid flow and its induced shear stress on the endothelial cells.^[^ ^17^, ^27^ ^]^ Static cultures may result in BBB models with lower expression of junction proteins, which ultimately affect the barrier structure and function, which, in turn, may lead to poor correlation with in vivo data.^[^ ^23^, ^28^, ^29^, ^30^, ^31^ ^]^ To improve the physiological relevance of in vitro models, dynamic in vitro BBBs featuring medium flow on the cell monolayer have been developed.^[^ ^17^, ^18^, ^20^, ^27^ ^]^ Medium perfusion enables improved delivery of oxygen and nutrients to the cells and provides physiological shear stress to the cells,^[^ ^32^ ^]^ which has been shown to greatly improve the tightness and function of BBB models in comparison to static culturing.^[^ ^23^, ^33^ ^]^ Furthermore, microfluidic‐based, 3D BBB models, which include multiple cell types that are cocultured in a 3D arrangement and often within a hydrogel matrix to better mimic the cellular microenvironment, have been reported in literature.^[^ ^17^, ^18^, ^21^, ^34^ ^]^ These systems are aimed at overcoming the limitations and low physiological relevance of culturing cells in planar arrangements by providing a 3D microenvironment, where different cell types can communicate, interact and exchange molecular cues with each other. Electrodes were also integrated into microfluidic devices to continually measure the transendothelial electrical resistance (TEER) of the barrier layer as a mean to reliably assess the formation, integrity, and permeability of the endothelial barrier without causing cellular and barrier damage.^[^ ^35^, ^36^, ^37^, ^38^ ^]^ TEER sensors have also been used for the evaluation of the integrity of barriers in 3D tissue models,^[^ ^39^, ^40^ ^]^ and transparent electrodes have been recently reported in literature.^[^ ^41^ ^]^ For reliable TEER evaluation, the uniformity of the current density across the cell layer is of fundamental importance.^[^ ^35^, ^37^ ^]^ This condition cannot be met by conducting TEER measurements using insertable, thin, chopstick‐like electrodes, which feature only low uniformity in current density. Moreover, electrode position may differ between measurements, which further compromises the reliability of the obtained TEER values.^[^ ^35^ ^]^ Microfluidic devices, featuring integrated TEER sensors with improved current‐density profiles, would provide a solution to overcome these limitations.^[^ ^35^, ^36^ ^]^

In this study, we developed an open‐microfluidic 3D BBB model and platform, which includes eight BBB devices per plate to perform parallel measurements under various conditions. Each BBB device consisted of a “brain” unit containing primary human astrocytes and human pericytes in a 3D hydrogel, to mimic the brain extracellular matrix (ECM), and a “vascular” microfluidic channel, which was lined with human cerebral endothelial cells. The on‐chip vascular unit was exposed to a gradually increasing, quasi‐unidirectional flow that was obtained by asymmetric periodic tilting of the BBB platform. The tilting‐induced flow promoted the formation of a robust and tight endothelial barrier layer. An integrated indium tin oxide (ITO)–platinum (Pt), four‐electrode TEER sensor enabled real‐time TEER and optical monitoring of the BBB. TEER measurements were used to monitor the formation and dynamic changes of the barrier, while the highly transparent ITO electrodes enabled unrestricted optical access to the barrier model for high‐resolution live imaging.

To evaluate the performance of the BBB system and to test for its ability to recapitulate barrier responses to traumatic events, we applied oxygen/glucose deprivation (OGD) conditions to mimic cerebral ischemia. We then investigated the rapid transformations that the BBB undergoes upon occurrence of such an insult. A key pathophysiological feature of cerebral ischemia is a rapid disruption of the BBB, however, its mechanism still remains elusive.^[^ ^5^, ^42^ ^]^ The limited understanding of ischemia‐induced BBB breakage impedes the development of therapeutic treatments for BBB stabilization, which are aimed at reducing brain edema and neurological damage.^[^ ^42^, ^43^, ^44^ ^]^ Our results show that the developed BBB‐on‐chip platform enables to recapitulate in vitro the rapid reorganization of the BBB and endothelial cell cytoskeleton and to monitor it continuously at high temporal resolution and spatial sensitivity.

## 2 Results

### 2.1 Device Design and Fabrication

**Figure 1:**
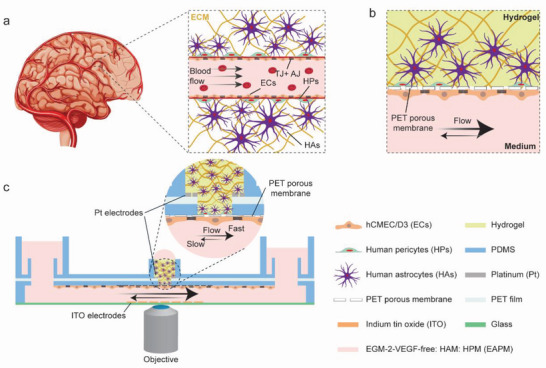
a) Schematic representation of the in vivo BBB, in which brain endothelial cells (ECs) line the blood vessels and are exposed to continuous blood flow. Human pericytes (HPs) and astrocytes (HAs) in the extracellular matrix (ECM) surrounded the vessels and, together with the ECs, formed the BBB. Tight junctions (TJ) between endothelial cells limited the transport of molecules across the barrier; b) schematic representation of the BBB on chip. ECs were seeded on a porous membrane to form a continuous, tight monolayer. Medium flow in the microfluidic channel exposed the ECs to shear stress. HAs and HPs were cultured in a 3D hydrogel structure on the opposite side of the porous membrane; c) illustration of the BBB chip and its operation. The ECs were cultured in the vascular microchannel, while HAs and HPs were cultured in the upper brain compartment. The coculture medium (EAPM, VEGF‐free EGM‐2:HAM:HPM = 1:1:1) was perfused through the microchannel by gravity‐driven flow to deliver oxygen and nutrients to the cells and to expose the EC layer to shear stress. High‐resolution microscopy could be carried out through the integrated ITO electrodes, while TEER values could be recorded using the on‐chip electrodes to measure barrier tightness.

The blood–brain barrier includes multiple cell types that jointly form a tight and selective barrier structure (**Figure** **1**a). To reconstitute the BBB structure on chip, we cocultured human cerebral microvascular endothelial cells (ECs), human astrocytes (HAs), and human pericytes (HPs) in a multilayer, open microfluidic device (Figure 1b). ECs were seeded along the walls of a microfluidic channel to form a monolayer structure and recapitulate a microvessel wall. The microchannel was flanked by two open reservoirs to generate gravity‐driven flow by tilting the platform, and it was separated from the brain compartment, where HAs and HPs were cultured, by a polyethylene terephthalate (PET) porous membrane. HAs and HPs in the brain compartment were embedded in a hydrogel matrix to provide physical support to the cells and recapitulate the 3D cellular arrangement. Nutrients in the brain compartment were delivered by transport and diffusion across the endothelial barrier structure and through the opening of the hydrogel/medium reservoir. The open‐microfluidic device enabled simple access for sampling on both sides of the cellular barrier to measure molecular transport and diffusion across the BBB. Integrated ITO and Pt electrodes on both sides of the barrier enabled the measurement of TEER values for real‐time monitoring of barrier formation and integrity. The transparent, integrated ITO electrodes were patterned on the bottom glass coverslip to enable live, high‐resolution confocal microscopy of the BBB structure and its reorganization (Figure 1c).

**Figure 2:**
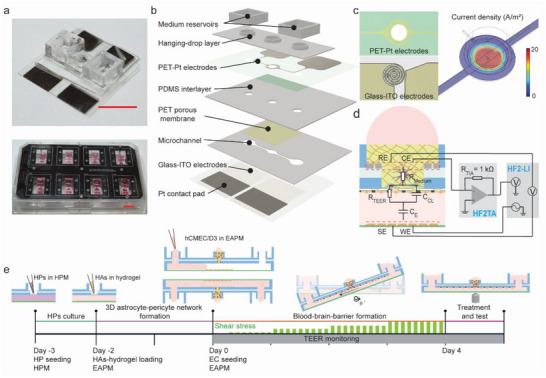
a) Photograph of the BBB chip (top) and the eight‐chip BBB platform (bottom). Scale bars = 1 cm; b) exploded view of the BBB chip, which consists of a glass‐ITO coverslip with patterned electrodes, a vascular microchannel layer, a PET porous membrane, a PDMS interlayer, a PET‐Pt electrode layer, the hanging‐drop layer and two open reservoirs; c) design of the on‐chip, integrated electrodes and FEM simulation of the current density at the EC layer. The glass‐ITO electrode was designed in a spiral shape to provide uniform current density at the BBB layer when combined with the top Pt ring electrode. The current density distribution was estimated by simulating a sinusoidal voltage stimulation of 200 mV_p_ amplitude at 1 kHz using COMSOL Multiphysics. The figure shows the current density at the EC layer; d) schematic design of the four‐electrode TEER measurement system and the electric‐equivalent circuit of the BBB (WE: working electrode, SE: sensing electrode, RE: reference electrode, CE: counter electrode), which includes the capacitance of the cell membranes (*C*_CL_), the resistance of the paracellular route (*R*_TEER_), the medium and hydrogel resistance (*R*_medium_), and the electrode capacitances (*C*_E_); e) experimental timeline of cell seeding and culturing on the BBB chips. On day −3, HPs were seeded on the porous membrane in the brain compartment, and the device was filled with pericyte medium (HPM). On day −2, HAs, which were previously resuspended in liquid hydrogel solution, were loaded into the brain compartment, and the medium in the device was replaced with the common medium EAPM. On day 0, the ECs were seeded into the microchannel. The platform was then turned upside down and operated in a hanging‐drop configuration to promote the sedimentation and the adhesion of the ECs onto the porous membrane. After 2 h, the platform was returned to a standing‐drop configuration, and asymmetric tilting was started to recirculate the medium and apply shear stress to the cell layer. The tilting angle and the resulting shear stress were gradually increased over the 4 d of culturing to obtain a tight cellular barrier. After the barrier had formed, different analyses and treatments were used to assess the integrity and functionality of the BBB model. TEER values were monitored and recorded during the whole EC‐barrier formation process.

Each BBB model was realized on a 26 mm × 25.5 mm × 4.2 mm (*L × W × H*) microfluidic device, and the overall BBB platform contained up to eight devices operated in parallel (**Figure** **2**a). The microfluidic network included a 100‐µm‐high microchannel with a central 3.5 mm‐diameter BBB region, which was connected to two open reservoirs at each end of the microchannel to realize the vascular unit, and a 3 mm‐diameter compartment to host the hydrogel matrix to host the brain compartment (Figure 2a,b and Figure S1, Supporting Information). A porous membrane separated the brain and the vascular side. Tilting of the chip generated a pulsed flow in the vascular channel to expose the ECs lining the channel walls and the porous membrane to shear stress. An asymmetrical tilting scheme was used to generate larger shear stress in one flow direction to avoid symmetric, bidirectional flow that could affect the endothelial layer.^[^ ^17^, ^45^ ^]^ The central BBB region on the vascular side was designed with a larger diameter with respect to the top brain compartment so as to expose the ECs forming the BBB to uniform shear stress and to avoid potential barrier leakage caused by lower shear stress along the channel edges (Figure S2, Supporting Information). Each system comprised a multilayer structure of glass–ITO, a thin poly(dimethylsiloxane) (PDMS) layer for microchannel fabrication, a porous PET membrane, an interconnecting PDMS layer to form the brain chamber, a Pt‐patterned PET foil, and a final PDMS layer featuring the medium reservoirs and circular rims for stable hanging‐drop operation during cell seeding (Figure 2b). Large Pt contact pads were patterned on the glass–ITO electrodes and on the top PET foil to provide reliable electrical connections and low overall electric resistance. Electrical connections between the printed circuit board (PCB) and the microfluidic devices were realized by spring‐loaded pins that contacted the electrode pads from above (Figure S3, Supporting Information). Up to eight chips could be mounted between a custom‐made PCB and the chip holder. This arrangement enabled to electrically interrogate the integrated TEER electrodes and holding the chips in position during culturing and imaging. The PCB and the chip holder featured standard well‐plate dimensions for compatibility with laboratory automation and microscopy tools. Open windows above and below the microfluidic networks provided optical access for widefield and confocal microscopy (Figure 2a; Figures S4 and S5, Supporting Information). The pump‐and‐tubing‐free operation of the chips promoted scalability and parallelization by simple stacking of multiple plates on a tilting stage.

To obtain a highly uniform current density at the barrier, a spiral‐shape bottom electrode was designed to compensate for the low current‐density uniformity arising from the top ring electrodes (Figure 2c and Figure S3, Supporting Information). We employed a four‐electrode measurement scheme to eliminate the resistance contributions of the connecting wires and traces and to deal with the high impedance of the double‐layer capacitance at the electrode–electrolyte interface. We performed frequency‐sweep measurements using a lock‐in amplifier to measure the current flowing through the system (voltage‐to‐current conversion by means of a transimpedance amplifier [TA]) and the differential voltage across the barrier model (Figure 2d and Figure S5, Supporting Information).

The on‐chip BBB was realized by loading the different cell types over multiple days (Figure 2e). First, HPs were loaded into the brain compartment and left to attach and grow on the porous membrane during 24 h. The following day, the HA‐hydrogel mixture was loaded into the brain compartment. The 3D astrocyte‐pericyte network formed within 2 d, then, the ECs were finally seeded into the microchannel. To promote adhesion of the ECs to the porous membrane, the chips were maintained in an inverted (hanging‐drop) configuration for 2 h. 3.5‐mm‐diameter circular hydrophobic rims were realized inside the medium reservoirs to generate a stable hanging‐drop network for inverted operation and to prevent medium spillage. After cell attachment, the chips were turned back into their standing‐drop configuration, and additional medium was added to the reservoirs to break the liquid drops and to wet the whole reservoir. This procedure was applied to avoid high Laplace pressures of small liquid drops, which would counteract the gravity‐induced pressure differences during tilting and reduce the flow rate during barrier formation. The culture medium was continuously recirculated between the two reservoirs of each chip by tilting the platform perpendicular to the microchannel axis. (Figure S6, Supporting Information), which provided shear stress to the ECs and increased the gas and nutrient exchange of the medium. The maximum shear stress, exerted on the ECs layer, was gradually increased over the culturing period by increasing the maximum tilting angle to +30° during the 4 d of EC culturing. The platform motion and tilting intervals were optimized to reduce medium backflow so as to generate directional flow across the ECs within physiological shear stress levels^[^ ^32^, ^46^ ^]^ (Figure S6, Supporting Information).

### 2.2 BBB Formation and Characterization

**Figure 3:**
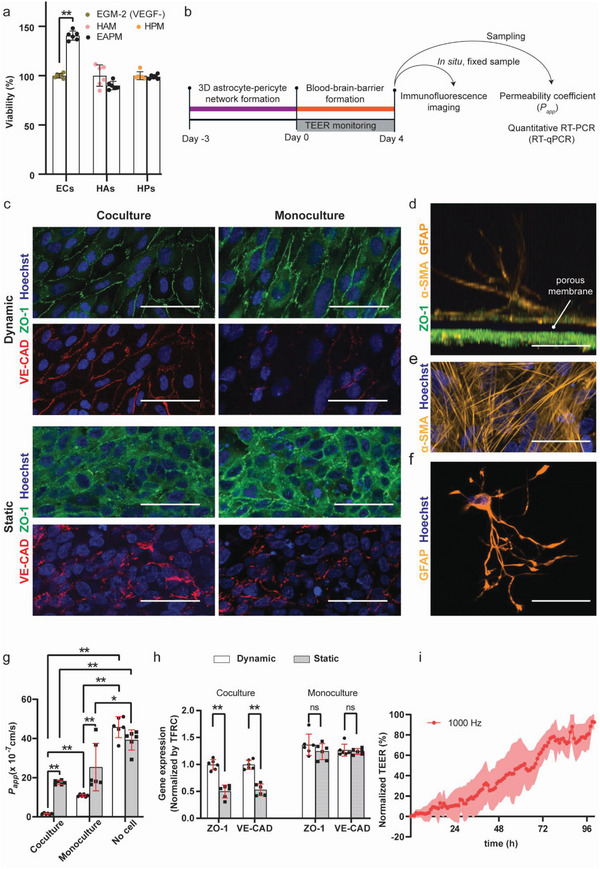
a) Cell viability in specific and common medium formulations (EGM‐2 (VEGF‐): VEGF‐free endothelial medium; HAM: human astrocyte medium; HPM: human pericyte medium; EAPM corresponds to EGM‐2‐(VEGF‐):HAM:HPM = 1:1:1; *n*  =  6 for each condition); b) schematic of the experimental workflow for the characterization of the BBB models; c) immunofluorescence microscopy images of the EC monolayer under different culturing conditions (maximum intensity projection, 3 µm z‐stacks). The monolayer was stained for the tight‐junction protein ZO‐1 (green), adherent‐junction protein VE‐CAD (red), and cellular nuclei (Hoechst, blue). For the dynamic culturing conditions, flow was directed along the vertical direction. However, in the barrier region, the flow had different directional components owing to the circular compartment and the widening of the channel; d) vertical cross‐section of the BBB model. Pericytes were cultured on the opposite side (top side) of the porous membrane with respect to the EC layer (below the porous membrane). The HPs were stained for *α*‐SMA (yellow) and nuclei (Hoechst, blue). Astrocytes were suspended in the 3D hydrogel matrix and stained for GFAP (yellow). e) Pericytes on the porous membrane; f) maximum intensity projection of an astrocyte on chip in the hydrogel matrix, showing that astrocytes presented the characteristic star‐shaped morphology in the hydrogel. g) Permeability coefficients (*P*_app_), calculated from the diffusion of 4 kDa FITC‐Dextran through a membrane with just hydrogel (No cell), an endothelial monolayer with hydrogel (Monoculture), an endothelial monolayer cocultured with HAs and HPs (Coculture), with and without the application of gravity‐driven medium flow (*n* = 6 for each condition); h) gene expression of ECs in monoculture and coculture under static and dynamic culturing conditions (*n* = 6 for each condition, all the data were normalized to the TFRC in the coculture system under dynamic conditions); i) normalized TEER values were measured using the integrated sensor every hour in the coculture system (the graph shows the mean values ± standard deviation in the form of a shaded envelope of the normalized TEER values at 1 kHz, *n*  =  6). Data represent mean values ± s.d., *p ≤ 0.05, **p ≤ 0.01; ns, not significant. Scale bars = 50 µm.

To investigate the efficiency of our system in supporting the growth of different cell types simultaneously, we measured the viability of ECs, Has, and HPs cells using either tissue‐specific medium formulation, namely, vascular endothelial growth factor (VEGF)‐free EGM‐2, human astrocyte medium (HAM), human pericyte medium (HPM), or a mixture of them, which we termed EAPM (VEGF‐free EGM‐2:HAM:HPM = 1:1:1). We confirmed that the mixed co‐culture medium formulation, EAPM, did not affect the viability of the different cell types in culture. After 4 d of culturing in EAPM, the cell metabolic activity of the HAs (91.28 ± 3.85%) and HPs (98.96 ± 1.98%) did not show any significant difference, in comparison to the viability in their specific medium formulations (**Figure** **3**a). In contrast, the viability of the ECs was significantly promoted in EAPM (138.75 ± 4.28%) in comparison to the VEGF‐free EGM‐2 (Figure 3b). The high viability of all cell types in the common medium formulation indicated that the cells could indeed be cocultured to reproduce a human BBB model on chip.

We then studied BBB tightness and EC monolayer formation under different culture conditions, namely dynamic (i.e., by perfusing via asymmetric, periodic tilting of the platform) and static culturing (i.e., by maintaining the platform in a horizontal configuration), and in monocultures of ECs or triple cocultures of ECs, HPs, and HAs. Barrier formation was characterized using real‐time TEER, end‐point permeability measurements, immunofluorescence staining, and protein expression via RT‐qPCR (Figure 3b). For all conditions, the ECs were cultured on chip during 4 d before characterization. Under dynamic culturing, the ECs showed an elongated morphology and orientation in the flow direction, with a higher localization of the tight‐junction protein zona occludens 1 (ZO‐1) and vascular endothelial cadherin (VE‐CAD) at the cell‐to‐cell junctions with respect to ECs under static conditions (Figure 3c). Coculturing with HAs and HPs further promoted the localization of ZO‐1 and VE‐CAD, an indication of tighter barrier formation.^[^ ^47^, ^48^ ^]^ Confocal microscopy imaging of the BBB structure confirmed that, under coculture conditions, the HPs grew on the opposite side of the porous membrane (Figure 3d,e) in close proximity to the EC monolayer, while HAs exhibited characteristic star‐shaped morphologies with extended end feet in the hydrogel matrix (Figure 3d,f and Figure S7, Supporting Information). These findings indicate that the BBB model featured an organized 3D multilayer cellular structure.

We assessed the barrier permeability by measuring the paracellular transport of 4 kDa FITC‐Dextran^[^ ^17^, ^18^ ^]^ under the different culturing conditions (Figure 3g). The BBB models cultured under static conditions, either as EC monoculture or triple coculture, did not show significant differences in permeability (*P*_app_ = 24.8 ± 1.4 × 10^−7^ cm s^−1^ and *P*_app_ = 17.3 ± 1.1 × 10^−7^ cm s^−1^, for monoculture and coculture conditions, respectively). However, their permeability was lower than that of the acellular control group (*P*_app_ = 38.6 ± 5.5 × 10^−7^ cm s^−1^), which suggests that a cellular barrier had formed on chip. Exposure to shear stress further decreased the permeability of the BBB models, which resulted in a twofold decrease for the monoculture BBB model (*P*_app_ = 10.5 ± 0.5 × 10^−7^ cm s^−1^) and a 12‐fold decrease for the coculture model (*P*_app_ of 1.4 ± 0.3 × 10^−7^ cm s^−1^). The application of shear stress, induced by the gravity‐driven flow, seemingly promoted the formation of a tighter barrier layer, which confirms the importance of the presence of shear stress in in vitro barrier models.^[^ ^32^, ^49^ ^]^ Furthermore, the permeability of the coculture barrier model was ≈7‐fold lower than that of the monoculture barrier with applied shear stress, which indicated that the coculture of ECs with HPs and HAs enhanced the tightness of the BBB model. Finally, the *P*_app_ of the dynamic, coculture BBB model was comparable to previously reported permeability values for both, in vitro (*P*_app_ ≈ 10^−6^–10^−7^ cm s^−1^)^[^ ^18^, ^50^, ^51^, ^52^, ^53^ ^]^ and in vivo (≈4 × 10^−7^ cm s^−1^ in rat)^[^ ^54^, ^55^ ^]^ BBB studies, which confirms that the quasi‐unidirectional, tilting‐induced medium flow effectively supported the formation of a tight barrier on chip.

Next, we performed RT‐qPCR to determine the expression of ZO‐1 and VE‐CAD, as markers of tight‐junction‐protein expression and cellular polarity (Figure 3h).^[^ ^18^, ^56^ ^]^ ECs in the coculture BBB models showed a lower expression of these proteins in comparison to their monoculture counterparts under static and dynamic conditions. This effect is particularly evident for the static coculture condition, which showed a much lower protein expression than the corresponding monoculture model. No significant difference in ZO‐1 and VE‐CAD expression was measured in EC monocultures under static or dynamic conditions. These measurements show that gene expression alone is not a good indicator of barrier tightness, as higher ZO‐1 or VE‐CAD gene expressions are seemingly not correlated to tighter cellular barriers.^[^ ^5^, ^57^, ^58^, ^59^ ^]^

Finally, the BBB formation of the coculture model under dynamic conditions was continuously monitored by using the integrated TEER sensor. The trans‐barrier resistance was recorded at multiple frequencies to improve the reliability of the detection (Figures S8 and S9, Supporting Information). To compare different chips, the TEER values were normalized between 0% (TEER at day 0, when ECs were loaded into the chip) and 100% at day 4 (Figure 3i). The normalization was necessary as different chips featured different baseline values owing to small variations in the alignment of the electrodes with the fluidic network during fabrication (Figure S10, Supporting Information). The continuous TEER measurements enabled us to follow and confirm the formation of a tight cellular barrier on chip.

### 2.3 Monitoring Rapid Disruption of the BBB

**Figure 4:**
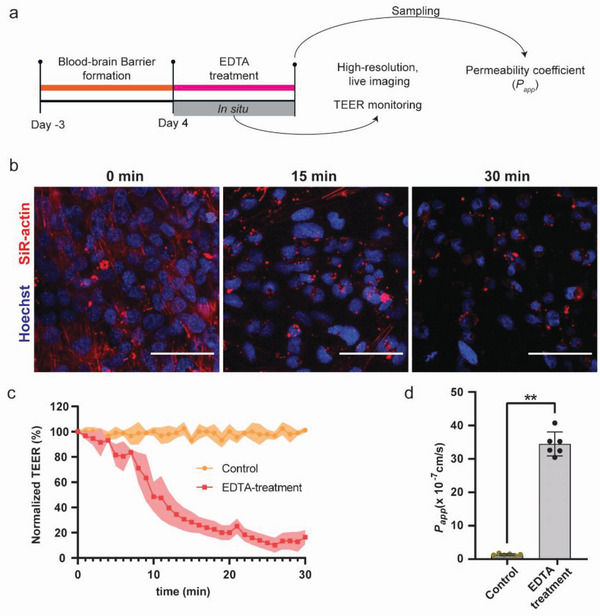
a) Schematic of the experimental workflow for monitoring EDTA‐induced barrier disruption; b) high‐resolution, live imaging of ECs on chip. The endothelial layer was stained for F‐actin (SiR‐actin, red) and cell nuclei (Hoechst, blue). EDTA induced actin‐fiber aggregation within 15 min and cell detachment of the barrier within 30 min of the treatment; c) TEER values were recorded every 2 min after EDTA dosage (the graph shows mean values ± standard deviation in the form of a shaded envelope of the normalized TEER values at 1 kHz, *n* = 6 for each condition); d) permeability measurements were acquired by measuring the diffusion of 4 kDa FITC‐Dextran after 30 min of EDTA treatment (*n* = 6 for each condition); Control group without EDTA treatment. Data represent mean values ± s.d., *p ≤ 0.05, **p ≤ 0.01. Scale bars = 50 µm.

We then sought to verify whether our coculture system could be used to monitor rapid variations in the BBB structure. To this end, we exposed the BBB to ethylenediaminetetraacetate (EDTA), a strong chelator of divalent cations, such as calcium (Ca^2+^) and magnesium (Mg^2+^), to induce cation depletion in the EC microchannel, which affects barrier tightness and promotes cell detachment.^[^ ^36^, ^60^, ^61^, ^62^ ^]^ To compare quantitative results obtained by TEER measurements with morphological and functional changes in barrier integrity, we simultaneously monitored focal adhesion and cell permeability disruption by high‐resolution time‐lapse microscopy. Barrier permeability was measured after EDTA treatment using the FITC‐Dextran fluorescent tracer (**Figure** **4**a).

Live imaging was performed by staining the cellular nuclei with Hoechst and the actin cytoskeleton with SiR‐actin. Within 15 min of exposure to EDTA, we observed the collapse of the actin filaments in the ECs, which indicates a loss of focal contacts of the ECs to the membrane support and a drastic variation in cell morphology,^[^ ^63^ ^]^ while cell detachment was observed after 30 minutes (Figure 4b). In parallel, the TEER value started to decrease as the EDTA solution was injected into the microchannel, showing a TEER decrease of 71.7 ± 8.3% of the initial TEER value within the first 15 min of EDTA exposure (Figure 4c). The rate of TEER decrease then reduced, and the TEER value stabilized at ≈12% of the original TEER value after ∼30 min. The pronounced drop of TEER upon EDTA application is in line with previously reported measurements in other organ‐on‐chip models.^[^ ^36^, ^60^ ^]^ Finally, we assessed the barrier permeability after 30 min of EDTA treatment to confirm these data. The *P*_app_ of the 4 kDa FITC‐Dextran was significantly increased, showing an ∼25‐fold increase after EDTA treatment (*P*_app_ = 34.8 ± 3.8 × 10^−7^ cm s^−1^) compared to the control condition (*P*_app_ = 1.4 ± 0.3 × 10^−7^ cm s^−1^; Figure 4d). These results demonstrate that our BBB platform is capable of real‐time monitoring—through TEER and high‐resolution microscopy—rapid variations in the in vitro BBB model upon external insults.

### 2.4 The BBB Platform as an Effective In Vitro Model for Recapitulating Ischemic Injuries

**Figure 5:**
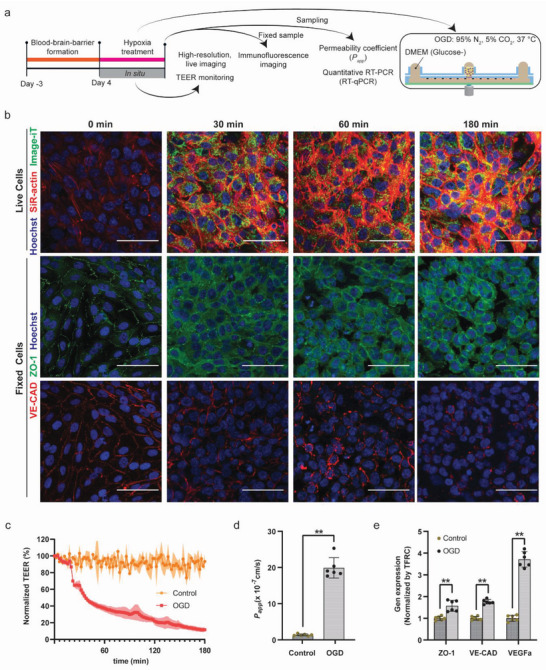
a) Schematic of the experimental workflow for recapitulating ischemic injury on chip; b) the cellular barrier on chips was imaged continuously, and separate chips were fixed for immunofluorescence image analysis after 0 min, 30 min, 1 h, and 3 h of OGD exposure. For live imaging, cells were stained for F‐actin (SiR‐actin, red), hypoxia (Image‐iT, green), and cell nuclei (Hoechst, blue). Large actin stress‐fiber aggregates appeared after 30 min of OGD exposure. After 1 h, cells started to assume a round morphology, which compromised barrier tightness. The effect was even more visible after 3 h of OGD insult. For fixed, immunofluorescent‐stained images, the EC layer was labeled for ZO‐1 (green), VE‐CAD (red), and cell nuclei (blue). After 30 min of OGD, ZO‐1 and VE‐CAD showed less localization at cell boundaries with respect to the initial barrier; at later time points, the barrier showed a drastic decrease in localization of the adherent and junction proteins, as well as a round cell morphology and openings and voids; c) TEER values were acquired every 2 min from chips exposed to hypoxia and control conditions after the OGD had been applied (graph shows mean values ± s.d. in the form of a shaded envelope of the normalized TEER values at 1 kHz, *n* = 6 for each condition); d) permeability measurements were carried out after 3 h of OGD exposure by measuring the diffusion of 4 kDa FITC‐Dextran across the barrier (*n* = 6 for each condition); e) gene expression of ECs under normal and OGD culture conditions, including the ZO‐1 tight junction protein, the VE‐CAD adherent junction protein and the vascular growth factors VEGFa; *n* = 6 for each condition). Control group without hypoxia treatment. Data represent mean values ± s.d., *p ≤ 0.05, **p ≤ 0.01. Scale bars: 50 µm.

After confirming that our system was capable of monitoring rapid variations in BBB permeability and EC‐layer organization, we challenged our barrier model with an ischemia‐like insult by exposing the model to oxygen glucose deprivation (OGD), which is a commonly applied method to induce ischemic injuries in vitro^[^ ^64^ ^]^ (**Figure** **5**a). OGD conditions were applied by replacing the EAPM by glucose‐free DMEM and by flushing the culture chamber with 95% N_2_–5% CO_2_.

We monitored the barrier behavior by continuously recording the TEER value and by using high‐resolution microscopy, for which we used live fluorescence markers for actin (SiR‐actin), cell nuclei (Hoechst), and cellular hypoxia (Image‐iT Green Hypoxia Reagent). Within 4 min of incubation under OGD conditions, the fluorescence signal of the hypoxia indicator appeared in the cellular barrier (Figure S12, Supporting Information), which indicated that our open‐microfluidic solution enabled us to quickly expose the BBB to hypoxia stress conditions. Concurrently, the cells started to show a marked increase in actin polymerization, as evidenced by the increased fluorescence of the actin filaments through the SiR‐actin staining. A strong SiR‐actin signal appeared within 30 min of OGD exposure, and actin stress fibers were clearly visible by using live, confocal microscopy. After 60 min of OGD exposure, the actin stress fibers started to clump together, which led to the contraction of the ECs, as clearly visible at 180 min post‐OGD exposure (Figure 5b; Videos S1 and S2, Supporting Information). The behavior of the ECs in our BBB devices confirmed that actin stress‐fiber polymerization caused an increase in cytoskeletal tension of the ECs, which led to an impairment of the junctions between ECs and to a hyperpermeability of the barrier.^[^ ^15^ ^]^

The cellular barriers were also fixed at different time points to inspect the barrier reorganization through immunofluorescence staining (Figure 5b). Within 30 min of OGD exposure, although the barrier remained intact, ZO‐1 and VE‐CAD showed less localization at cellular junctions, which indicated that the junction proteins quickly rearranged under OGD. After 60 min of OGD exposure, the cellular barrier showed clear signs of disruption, and the ECs started assuming a rounder morphology. After 180 min of OGD exposure, the barrier was completely compromised, showing holes and void spaces between the cells.

The continuous TEER measurements showed that the TEER value started decreasing 6 min after exposure to OGD, indicating hypoxia‐stress‐induced barrier dysfunction before actin polymerization could be observed through live imaging (Figure 5c; Figure S12, Supporting Information). TEER decreased by 6.7 ± 1.8% within the first 10 min, and, then, the value rapidly dropped by 35.7 ± 6.7% within 30 min of exposure. Eventually, the TEER value dropped by 88.4 ± 2.4% within 3 h of the OGD insult, which indicated the disruption of the barrier on chip. We subsequently measured the permeability of the barrier using the 4 kDa FITC‐Dextran tracer to further evaluate the tightness of the barrier on the chips (Figure 5d). In accordance with the TEER measurements, the *P*_app_ was significantly increased by ∼20 fold after 3 h of OGD exposure (22.2 ± 4.4 × 10^−7^ cm s^−1^) compared to the control condition (1.2 ± 0.1 × 10^−7^ cm s^−1^). The TEER and permeability measurements confirmed that the barrier was disrupted by the OGD insult. We also measured the activity of the P‐glycoprotein (P‐gp) transmembrane efflux pump of the endothelial cells, which is known to be increased after an ischemic insult,^[^ ^65^, ^66^ ^]^ after OGD exposure by detecting the accumulation of Rhodamine 123 in the endothelial cells (Figure S13, Supporting Information). The results showed a drastic reduction of Rhodamine 123 in the EC layer after OGD exposure, which indicated an increased activity of the efflux pump as a consequence of the OGD insult.

RT‐qPCR analysis of the harvested ECs after 3 h of exposure to OGD conditions showed that OGD led to significantly increased expression of ZO‐1 (1.57 ± 0.25), VE‐CAD (1.76 ± 0.10), and VEGFa (3.71 ± 0.36) compared to normoxic control samples (Figure 5e). These results suggest a compensatory mechanism for the reduction of tight junctions to counteract barrier disruption.

## 3 Discussion

In this work, we described the development and validation of a microfluidic platform with an integrated transparent TEER sensor to model the BBB in vitro. The platform has the following advantages over previously reported in vitro BBB models: 1) it combines a 2D endothelial monolayer with a 3D brain microenvironment to reconstruct the BBB structure on chip; 2) it features a tubing‐ and pump‐free fluidic system to induce flow across the endothelial layer, which enables straightforward parallelization and provides increased throughput; 3) it features a quasi‐unidirectional, gravity‐driven flow that provides shear stress at physiological levels; 4) it includes an open‐microfluidic network for simple liquid exchange and sampling; 5) it contains integrated TEER electrodes, which were optimized for uniform current density across the cellular layer; and 6) the integrated ITO electrodes are fully transparent and enable simultaneous high‐resolution imaging and TEER measurements.

TEER measurements are a standard, noninvasive method for evaluating barrier tightness in in vitro models, and both, integrated^[^ ^23^, ^36^, ^38^ ^]^ or plug‐in wire electrodes^[^ ^13^, ^18^, ^20^, ^51^, ^67^ ^]^ have been used in literature to monitor the characteristics of microfluidics‐based BBB models. Wire electrodes can be easily integrated or inserted on demand within microfluidic systems, without requiring specialized and complex fabrication steps. However, this type of electrode usually does not provide uniform current density across barrier layers due to the wire geometry. For non‐integrated electrodes, differences in electrode placement between measurements add an additional source of variation to the TEER measurements. The placement of electrodes at different locations has recently been used to obtain spatially resolved information about the uniformity of the cell coverage of a barrier model.^[^ ^68^ ^]^ However, to monitor variations over time, such an approach would require to repeatedly place the electrodes at precisely the same locations. Moreover, plug‐in wire‐electrodes, which are typically inserted into the inlet and outlet of the microfluidic device and distant to the cellular barrier, may lead to strong artifacts in TEER measurements due to resistance contributions of the cell culture medium in the microfluidic system containing the electrodes and the cellular barrier.^[^ ^35^, ^38^, ^69^ ^]^ Therefore, the sensitivity and the reliability of TEER measurements and the corresponding information on the tightness of the whole barrier may be compromised.^[^ ^35^ ^]^ To obtain sensitive TEER measurements, thin‐film TEER sensors have been integrated within microfluidics‐based systems^[^ ^23^, ^36^, ^70^, ^71^ ^]^ using different electrode materials (e.g., Au, Ag, Pt) on a variety of substrates (e.g., glass, PET, PC). Although electrodes, realized through metal films of a few tens of micrometer thickness, have been used as partially transparent electrodes for visible light,^[^ ^36^ ^]^ it has to be noted that metal films do not enable unhindered optical access for high‐resolution microscopy. Moreover, imaging could only be performed as an end‐point examination after disassembling the platform. As an alternative, electrodes that did not extend across the whole barrier region have been used, which resulted in non‐uniform current densities across the barrier layer. Recently, transparent electrodes have been used to monitor the integrity of a monolayer intestinal‐barrier model in a closed microfluidic chip, providing optical access to the barrier for wide‐field microscopy.^[^ ^40^ ^]^ However, the TEER recordings were performed using a two‐electrode setup, in which the double‐layer capacitance at the electrode–electrolyte interfaces contributed to the overall measured impedance of the barrier model. Here, we overcame such limitations by integrating transparent, thin‐film TEER sensing electrodes on glass coverslips in close proximity to and on both sides of the cellular barrier and by implementing a four‐electrode readout system. This approach enabled to reduce the contribution of the electrical resistance of the cell‐culture medium and helped to avoid measurement fluctuations as a consequence of varying sensor placements during multi‐day measurements. Through FEM simulations, we optimized the design of the electrodes so as to obtain a uniform current density across the barrier layer. The electrode geometry was designed to enable the use of open‐microfluidic structures to provide direct access to the “brain” side of the BBB model for simple loading of the hydrogel matrix for 3D cell culturing and for the sampling of the supernatant. We found that a spiral‐shaped electrode, in combination with a ring electrode for the microfluidic open access, provided uniform current density across the barrier layer. Although the electrodes were located within the optical path to the barrier layer, the high transparency of the ITO electrode and the use of a microscope coverslip as substrate material ensured compatibility with live, high‐resolution, confocal microscopy, so that rapid variations in the organization of the cellular barrier layer could be observed at high temporal resolution and with uniform spatial sensitivity.

In a first step in the realization of our in vitro BBB model, we confirmed that a common medium formulation could be used for coculturing ECs, HAs, and HPs in the platform. Our results showed that all cell types had a viability of more than 85% in the EAPM coculture medium, with ECs even presenting higher viability than in their standard medium despite the removal of VEGF to improve barrier tightness. We reproduced the BBB arrangement on chip by combining a 2D endothelial monolayer with a 3D brain‐like culture microenvironment for the HAs and HPs. In vivo, HPs surround the endothelial microvessels and fundamentally affect the function of the BBB in combination with the HAs.^[^ ^72^, ^73^ ^]^ In our model, the HPs were cultured in close proximity to the EC layer and within the same hydrogel compartments as the HAs to support the structure and function of the BBB cellular barrier on chip. The selected hydrogel matrix for culturing of HAs in 3D supported the growth of HAs that displayed long branches and end feet in close contact with the HPs (Figure S7, Supporting Information), which contributed to the formation of a robust and functional BBB on chip.^[^ ^18^, ^74^ ^]^

In vessels, blood flow generates shear stress on the endothelial cell layer, which is known to maintain the structure and function of the vessel barrier layer by regulating endothelial cytoskeleton and vascular homeostasis.^[^ ^32^, ^75^ ^]^ To recapitulate this effect in vitro, several BBB models have relied on external pumping systems to generate medium flow inside microfluidic devices and to improve the tightness of the barrier model.^[^ ^18^, ^20^, ^21^, ^50^, ^67^, ^76^ ^]^ However, tubing and pump systems impede parallelization for medium‐to‐high‐throughput studies due to the complex experimental setup that is required to operate each BBB model.^[^ ^17^ ^]^ Here, the medium flow was achieved by tilting the platform^[^ ^77^, ^78^ ^]^ without the use of external tubing and pumping devices. Although the generated flow was not perfectly unidirectional, as would have been the case for using an external pump, our results show that the asymmetric tilting profile and microfluidic‐chip geometry enabled to generate shear stress with a preferential directionality and within the physiological range, which improved the integrity and functionality of the in vitro BBB model. Our permeability measurements and immunofluorescence images show that the BBB, cultured under perfusion conditions, formed a tighter barrier, with localized junction and adherent proteins between ECs in the barrier, in comparison to static culture conditions. Moreover, ECs showed an improved orientation along the flow direction, while ECs in static conditions presented a round morphology.

In the monoculture system, we did not see a significant difference in the expression of junction‐specific proteins between conditions with and without shear stress, however, the permeability of the barrier was significantly lower under dynamic culturing conditions compared to static culturing. These observations may indicate that the shear stress does not affect the expression of junction‐specific proteins at the mRNA level, but promotes the specific localization of such proteins, as has been previously reported for human iPSC‐derived endothelial cells.^[^ ^79^ ^]^ In the coculture system on our chips, the expression of these junction‐specific proteins was slightly downregulated under perfusion conditions in comparison to the monoculture on chip. However, the dynamic, coculture BBB model exhibited a significantly lower permeability, which evidences the importance of coculturing ECs with HAs and HPs and of using a dynamic culturing environment to promote junction‐protein localization at cell–cell boundaries in the EC layer to achieve the formation of a tight barrier.^[^ ^80^ ^]^ The formation of a tight on‐chip barrier was further confirmed by the continuous increase in TEER value during EC culturing, which was detected by the integrated sensor.

After validating the BBB model, we investigated whether the integrated transparent TEER sensor could be used to monitor rapid variations in the cellular barrier upon exposure to external insults, concurrently with high‐resolution microscopy. To this end, we exposed the BBB model to EDTA, which is known to disrupt the junction protein assemblies and promote cell detachment.^[^ ^60^, ^62^ ^]^ The EDTA exposure caused a rapid breakage of the barrier, which, in turn, resulted in a rapid drop in the TEER values, while morphological changes and loss of focal adhesion of the ECs, as well as cellular detachment from the porous membrane, were detected via high‐resolution microscopy. The EDTA test confirmed that the integrated TEER sensor was able to detect and quantify a rapid variation in barrier integrity, which could be simultaneously confirmed through live, confocal microscopy.

Finally, we used the BBB platform to recapitulate the disruption of the BBB on chip by an ischemic insult upon applying oxygen‐glucose‐deprivation conditions. The TEER values rapidly decreased within the first few minutes after OGD onset. The OGD triggered actin polymerization in the ECs to induce cytoskeletal alteration, which is known to be an initiator of BBB rupture.^[^ ^15^, ^81^ ^]^ After ≈30 min, live imaging revealed that actin stress fibers were continuously being formed within the EC layer, which produced contractile forces in the cells, with the actin cytoskeleton directly pulling on the adherent and tight junction proteins VE‐CAD and ZO‐1.^[^ ^81^ ^]^ Gene‐expression analysis revealed that these adherent and junction proteins were highly expressed under hypoxia conditions in comparison to the normoxia samples, which may indicate that the EC layer tries to maintain its integrity by promoting the expression of the cellular junctional protein to counteract the cytoskeletal contractile forces. Live and immunofluorescence microscopy images showed that excessive stress‐fiber tension ultimately led to EC contraction, which resulted in the disruption of junctional protein assemblies and the subsequent removal of VE‐CAD and ZO‐1 from cell–cell contact boundaries as well as their internal redistribution.^[^ ^15^, ^81^, ^82^ ^]^ The activity of the P‐gp transmembrane efflux pump was also markedly increased after OGD exposure as previously reported in both, in vitro and in vivo ischemic studies.^[^ ^65^, ^66^ ^]^ Our model also confirmed the overexpression of VEGFa due to hypoxic stress, which has been hypothesized being a defense mechanism of the BBB during ischemia, through which the vessels promote angiogenesis to respond to barrier‐induced OGD stress.^[^ ^83^, ^84^ ^]^ TEER value decreases were evident before the appearance of the large stress‐fiber assemblies and cell contractions, which indicates that a leaky barrier had formed before drastic morphological changes in the EC layer occurred. This finding demonstrates the high sensitivity of TEER as a real‐time monitoring method for barrier tightness and the advantages of performing concurrent TEER and microscopy measurements to investigate barrier‐disruption events.

## 4 Conclusion

In summary, we established a robust, highly integrated, and user‐friendly BBB model platform that can be used to recapitulate barrier functions and to investigate rapid variations of the endothelial layer in response to external insults. Using the BBB platform to mimic cerebral ischemia, the model can contribute to elucidating the mechanism involved in barrier disruption, which will facilitate the discovery of BBB stabilizers to reduce brain edema and neurological damage. In addition, the developed BBB platform provides a tool for the development of therapeutic agents for brain and BBB‐related diseases. The high level of tightness of the BBB renders the developed system a suitable in vitro tool for screening novel therapeutic compounds and strategies for BBB crossing, a major roadblock in the development of compounds for CNS diseases. Moreover, the combination of an open‐microfluidic, hanging‐drop BBB platform will provide the possibility of easily integrating preformed brain‐disease tissue models into the system to investigate both, the barrier permeability to candidate compounds as well as compound efficacy on the target tissue.

## 5 Experimental Section

### Platform Fabrication

The multilayer microfluidic device was designed by using Inventor software (Autodesk, München, Germany). The device featured: one PDMS (Sylgard 184, Dow Corning Corp., Midland, MI) microchannel layer that was plasma bonded (PDC‐002, Harrick Plasma, New York, USA) to a 1.5# glass coverslip, which had been patterned with ITO electrodes (glass–ITO, 30–60 Ω, Diamond Coatings, West Midlands, UK); a semipermeable PET membrane with a pore size of 0.4 µm and a thickness of 12 µm (it4ip, Louvain‐la‐Neuve, Belgium); one PDMS interlayer; a platinum‐patterned PET (Sigma‐Aldrich, Buchs, Switzerland) electrode layer; a PDMS hanging‐drop and medium‐reservoir layer. As it is difficult to completely remove the hydrogel and cells from the microfluidic channel and brain compartment, this study opted for disposing off the devices after use. Detailed information on the platform fabrication and assembly is reported in the Supporting Information.

### Cell Culture

Human cerebral microvascular endothelial cells (hCMEC/D3, Tebu‐Bio GmbH, Offenbach, Germany) were cultured in Endothelial Growth Medium (EGM‐2, Lonza, Basel, Switzerland) in a cell‐culture flask, which was previously coated with 150 µg mL^−1^ collagen type I (collagen I, Roche, Basel, Switzerland). All experiments were performed with hCMEC/D3 (ECs) between passage numbers 36 and 39. HAs (ScienCell, Carlsbad, USA) and HPs (ScienCell, Carlsbad, USA) were cultured in 20 µg mL^−1^ poly‐l‐lysine (PLL, Sigma‐Aldrich, Buchs, Switzerland) coated flasks and maintained in HAM (ScienCell, Carlsbad, USA) and HPM (ScienCell, Carlsbad, USA), respectively. The HAs and HPs were used at passage numbers 3–6. All cells were maintained at 37 °C and 5% CO_2_ inside a cell‐culture incubator. The medium was replaced every 2 d. HAs and HPs were detached using 0.05% trypsin/EDTA (Gibco, Thermofisher Scientific, Reinach, Switzerland) and hCMEC/D3 were detached using 0.25% trypsin/EDTA (Gibco, Thermofisher Scientific, Reinach, Switzerland).

For barrier formation, EGM‐2 without VEGF was used, as VEGF was shown to increase the permeability of endothelial cell monolayers.

The viability of the different cell models under different medium conditions was quantified using a Cell Counting Kit‐8 assay (CCK‐8, Sigma‐Aldrich, Buchs, Switzerland). ECs, HAs, and HPs were seeded in VEGF‐free EGM‐2, HAM, and HPM, respectively, in a collagen I‐ or PLL‐coated 96‐well plate at a density of 1 × 10^4^ cells/well. After 48 h of culturing under the respective medium and coating conditions, the medium of six wells of each cell type was changed to the EAPM mixture (VEGF‐free EGM‐2:HAM:HPM = 1:1:1, 2% FBS). After 96 h of culturing, 10 µL of CCK‐8 reagent were added to each well and incubated in a well plate for 4 h in a cell culture incubator. The optical absorbance of each sample at 450 nm was measured using a Tecan plate reader (Infinite M1000 PRO, Tecan Trading AG, Männedorf, Switzerland).

### Cell Culture in the Device

At day ‐3, the microfluidic devices were sterilized by exposure to ultraviolet (UV) light for 30 min and mounted into the holder and assembled with the PCB. The microchannel and the brain compartment of each chip were coated with 500 µg mL^−1^ PLL for 2 h in a cell‐culture incubator to improve the adhesion of the hydrogel in the compartments and to promote cell attachment on the porous membrane. The PLL solution was then removed, and the channels and compartments were rinsed with and stored in DI water.

To form a 3D astrocyte‐pericyte network, DI water was removed from the chips, and 15 µL of human pericyte suspension at a concentration of 2 × 10^4^ cells mL^−1^ in HPM were loaded into the brain compartment of each chip. The microchannel was also filled with the HPM from the side reservoirs. The HP‐loaded devices were placed into a cell culture incubator for 2 h in a static configuration to allow for the HPs to sediment and to attach to the porous membrane. Then, the non‐adherent cells were removed from the chips by gently replacing the medium in the brain compartment in the hanging‐drop configuration.

The next day, the medium on the chips was removed, and 10 µL of the hydrogel‐HA mixture was loaded into the brain compartment of each chip. The hydrogel was prepared by mixing Gamma 2‐RGD (Manchester BIOGEL, Cheshire, UK) and collagen I (Advanced Biomatrix, Carlsbad, USA). Prior to mixing, the Gamma 2‐RGD was diluted 2.5 times in Peptisol (Manchester BIOGEL) to reduce the hydrogel stiffness. The diluted Gamma 2‐RGD was then mixed with collagen I solution to reach a final collagen concentration of 1.6 mg mL^−1^. HAs were then resuspended in the liquid hydrogel mixture at a concentration of 1 × 10^4^ cells mL^−1^. After loading the hydrogel‐HA mixture, the microchannels were filled with EAPM, and the chips were placed in the cell‐culture incubator for 10 min in a static configuration to allow for the solidification of the hydrogel. Then, the medium in the microchannel was refreshed with prewarmed EAPM, and 20 µL of prewarmed EAPM were loaded into the brain compartments on the hydrogel. The chips were then placed in the incubator, and the medium was exchanged 3 times within the first hour to balance the ion concentration in the hydrogel, as per the manufacturer instructions. The cells were afterwards cultured for 2 d on chip to form a 3D astrocyte‐pericyte network.

At day 0, the medium in the microchannels was removed, and the channels were washed three times with prewarmed phosphate‐buffered saline (PBS). The microchannels were then filled with a 200 µg mL^−1^ collagen I solution for 30 min to promote EC attachment in the microchannels and on the porous membranes. The microchannels were rinsed with prewarmed DI water twice. Subsequently, 80 µL of hCMEC/d3‐cell suspension at a concentration of 1 × 10^7^ cells mL^−1^ in EAPM were loaded into the microchannels of each device. The devices were immediately turned upside down to achieve a hanging‐drop configuration and incubated for 2 h in the cell‐culture incubator to allow for cell sedimentation and attachment to the PET membrane. After 2 h, the non‐adherent cells were removed by rinsing the microchannels with fresh EAPM. Afterward, the platform was placed in a standing‐drop configuration on a programmable tilting stage (InSphero AG, Schlieren, Switzerland) in the incubator. The tilting angle was increased gradually every day to allow for barrier formation (Figure S6, Supporting Information). Medium exchange was performed daily.

### TEER Measurements

TEER measurements were performed using an HF2‐LI lock‐in amplifier (Zürich Instruments AG, Zürich, Switzerland). The microfluidic devices were contacted via the custom‐made PCB to route the connections from the lock‐in amplifier to the integrated electrodes. A custom‐made LabVIEW (National Instruments, Austin, USA) user interface was used to control the selection of the microfluidic device and to control signal acquisition. Signal routing on the PCB was performed by the on‐board ADG1407 multiplexers (Analog Devices, USA).

A sinusoidal AC voltage signal with 200 mV peak voltage and central frequencies ranging from 500 Hz to 20 kHz was applied to the working electrode (WE) of the selected microfluidic device, while the CE was kept at pseudo‐ground by the HF2‐LI transimpedance amplifier (HF2TA, Zürich Instruments AG). The current flowing through the CE, i.e., across the BBB model, was converted to voltage using the HF2TA with a 1‐kΩ feedback resistor and sampled by the HF2‐LI. Differential voltage measurements between the vascular and brain compartments were acquired via the sensing electrode (SE) and the reference electrode (RE), in close vicinity to the current‐injection WE and CE, respectively. Voltage and current measurements were then used to calculate the resistance of the barrier layer.

The TEER sensor was characterized by sweeping the carrier frequency from 12.5 Hz to 20 kHz. The recorded signal at 12.5 Hz was ≈15 times lower than at 1 kHz resulting in a much lower signal‐to‐noise ratio and lower reliability of the recorded signal (Figure S8, Supporting Information). For this reason, this study opted for sweeping the signal frequency only in the frequency range of 500 Hz to 20 kHz during on‐chip barrier formation. For TEER value calculation, the 1 kHz measurements were selected (frequency sweeps and TEER recordings including impedance and phase results are shown in Figure S9, Supporting Information).

The initial resistance values (*R*_0_) were measured on day 0 before the ECs were seeded and represented the background resistance the system. All the resistance data (*R*_TEER_) were calculated by subtracting the baseline resistance value (*R*_0_) from the measured resistance data (*R*_m_) and then normalized with respect to the permeable area of the microchannel and, finally, converted to TEER values (Ω cm^2^) using the following equation (Figure S9, Supporting Information)

(1) $$\mathrm{TEER}=R_{\mathrm{TEER}}\times A=\left(R_{m}-R_{0}\right)\times A$$

where *A* indicates the permeable area of the microchannel in cm^2^.

The alignment of the electrodes with the fluidic network was performed manually, which resulted in differences in baseline resistances and TEER sensitivity among different chips. Therefore, TEER values were normalized between 0% (baseline TEER at day 0, before loading of the ECs) and 100% (TEER value at day 4) to compare different chips and to evaluate the formation and the disruption of the barriers.

### Permeability Measurement

4 kDa FITC‐Dextran (Sigma‐Aldrich) were used as a fluorescent tracer to assess the permeability of the BBB models. After 4 d of EC culture in the devices, 200 µg mL^−1^ of FITC‐dextran in EAPM were loaded into the microchannel, while fresh EAPM was loaded into the hanging‐drop compartment. After 5 h of incubation, either in static or dynamic mode according to the BBB model under investigation, 7 µL of medium aliquots were sampled from each microchannel and from the brain compartments. The aliquots were then pipetted in a 384‐well plate (Flat Bottom Black Polystyrene, Greiner Bio‐One, Gallen, Switzerland) prefilled with 7 µL EAPM. The fluorescence intensity of each sample aliquot at 520 nm was measured with a Tecan microplate reader (490 nm excitation wavelength, 520 nm emission wavelength). A standard curve with different concentrations (0–200 × 10^−6^ m) of FITC‐Dextran in EAPM was also acquired on the same measurement plate to quantify the concentration of fluorescence tracer in each sample well (Figure S11, Supporting Information). Control measurements using microfluidic chips without cells were also acquired for each experimental set.

The apparent permeability coefficient (*P*_app_) was calculated by using the following equation

(2) $$P_{\mathrm{app}}=\frac{V_{\mathrm{HD}}\times C_{\mathrm{HD}}}{A\times t\times \left(C_{\mathrm{MC}}\times V_{\mathrm{MC}}+C_{\mathrm{HD}}\times V_{\mathrm{HD}}\right)/\left(V_{\mathrm{MC}}+V_{\mathrm{HD}}\right)}$$

where *P*_app_ is the permeability coefficient, *t* is the time of incubation in seconds, *V*_HD_ is defined by the volume of medium in the hanging‐drop compartment, *C*_HD_ is the measured concentration of FITC‐dextran in the hanging‐drop compartment at incubation time *t*, *V*_MC_ is the volume of the microchannel, *C*_MC_ is the measured concentration of FITC‐dextran in the microchannel at time *t*, and *A* is the permeable area of the microchannel in cm^2^.

### EDTA Treatment and Live Imaging

EDTA (Thermofisher Scientific) was used to disrupt the BBB after 4 d of EC culture in the devices to validate the on‐chip TEER sensor and test the live‐imaging capabilities of the device in detecting rapid variations in EC‐layer organization. Before exposing the cells to EDTA, the culture medium in the microfluidic devices was changed to EAPM with 1 × 10^−6^ m SiR‐actin (Spirochrome AG, Schaffhausen, Switzerland) and 2 drops mL^−1^ Hoechst (Hoechst 33342 NucBlue staining solution, Invitrogen, Thermofisher Scientific) to fluorescently stain the cells for live imaging. After 1 h of incubation, the platform was transferred to a confocal microscope to observe the behavior of the ECs in situ by simultaneous live, confocal imaging and real‐time TEER detection. Subsequently, the medium in then microfluidic chips was replaced by a prewarmed 8 × 10^−3^ m EDTA‐PBS solution. Imaging and TEER‐measurement intervals were set to 2 min.

Live imaging was carried out using a Nikon spinning‐disk confocal microscope (W1‐Sora, Nikon, Egg, Switzerland) with environmental control for live‐cell imaging using a 40× water‐immersion objective. Image processing was done using the NIS‐Elements software package (Nikon, Egg, Switzerland).

### OGD Treatment and Live Imaging

After 4 d of EC culturing in the devices and prior to performing OGD studies, the culture medium in the microfluidic devices was changed to EAPM with 5 × 10^−6^ m of Image‐iT Green Hypoxia Reagent (Thermofisher Scientific), 1 × 10^−6^ m SiR‐actin, and 2 drops mL^−1^ Hoechst to stain the cells for live‐imaging. After 1 h of incubation, OGD conditions were applied by changing the medium in the chip to Dulbecco's modified Eagle medium, with no glucose (DMEM (glucose‐free), Gibco, Thermofisher Scientific) and by reducing the O_2_ level to 0% by placing the platform into an environmental chamber on the confocal microscope stage, which was flushed with 5% CO_2_, 95% N_2_ and maintained at 37 °C. Live imaging was performed using a Nikon spinning disk confocal microscope with a 40× water‐immersion objective. Imaging and TEER‐measurement sampling intervals were set to 2 min. Image processing was done using the NIS‐Elements software package.

### P‐gp Activity On‐Chip

To assess P‐gp activity in the BBB model, the culture medium in the microfluidic devices was changed to DMEM (glucose‐free) or EAPM with or without 5 × 10^−6^ m of the P‐gp inhibitor Verapamil (Verapamil hydrochloride, Sigma‐Aldrich, Buchs, Switzerland) under OGD or normal conditions. After 30 min of incubation, 5 × 10^−6^ m of P‐gp substrate Rhodamine 123 (Rho 123, Sigma‐Aldrich, Buchs, Switzerland) in (glucose‐free) medium were injected into the microfluidic devices after medium exchange. The platform was incubated for 2.5 h under OGD or normal conditions, and the Rhodamine 123 was subsequently removed and replaced by normal medium. Rhodamine 123 uptake of the ECs was imaged using a Nikon spinning‐disk confocal microscope with a 40× water‐immersion objective. Image processing was carried out using the NIS‐Elements software package.

### Immunofluorescence and Confocal Imaging

For sample fixation, the cells in the devices were washed with PBS and fixed in intracellular (IC) fixation buffer (Sigma‐Aldrich) for 20 min at room temperature (RT), followed by 20 min of 0.1% Triton X‐100 (Sigma‐Aldrich). After washing and blocking with 5% (w/v) bovine serum albumin (BSA, Sigma‐Aldrich) for 2 h at RT, conjugated antibodies against the tight‐junction protein ZO1‐1A12 (conjugated with Alexa Fluor 488, 1:200 dilution, Thermofisher Scientific), the intercellular adherent protein VE‐cadherin (VE‐CAD, conjugated with Alexa Fluor 647, 1: 50 dilution, BD Biosciences, Allschwil, Switzerland), glial fibrillary acidic proteins (GFAP, conjugated with Cy3, 1:200 dilution, Merck Millipore, Schaffhausen, Switzerland), and alpha smooth muscle actin (*α*‐SMA, conjugated with Cy3, 1:400 dilution, Merck Millipore) were added to a 0.1% (w/v) BSA solution in DPBS and incubated at 4 °C overnight. After washing the samples with PBS, the cells were incubated with the nuclear stain Hoechst for 30 min at RT and stored in PBS at 4 °C until image acquisition. Immunofluorescence images of the barrier were acquired with a Nikon spinning disk confocal microscope using a 40× water‐immersion objective. Image processing was done using the NIS‐Elements software package.

### Real‐Time Quantitative PCR (RT‐qPCR)

To compare the relative gene expression levels among different treatment and culturing groups, samples for RT‐qPCR analysis were generated by harvesting ECs cultivated on chips in mono‐ or coculture, static or under shear stress, and with or without hypoxia treatment (*n* = 3 technical replicates and *n* = 3 biological replicates). RNA isolation was performed with the NucleoSpin RNA XS kit according to the manufacturer's instructions (Machery‐Nagel, Oensingen, Switzerland). For complementary deoxyribonucleic acid (cDNA) synthesis, the isolated RNA was reversely transcribed into a 20 µL total volume using the RNA to cDNA EcoDry Premix (Oligo dT) (TaKaRa, Saint‐Germain‐en‐Laye, France) according to the manufacturer's instructions. RT‐qPCR was conducted using a QuantStudio 3 (Applied Biosystems, Thermofisher Scientific, Reinach, Switzerland), and primers were purchased from Integrated DNA Technologies (IDT, Zürich, Switzerland). The gene expression assays with human‐specific primers for tight junction protein ZO‐1 (TJP1), adherent junction protein VE‐cadherin (CDH5), vascular endothelial growth factor A (VEGFa), TFRC (CD71) were used for gene expression analysis in human ECs (Table S1, Supporting Information). X‐fold changes in the relative mRNA expression were determined using the comparative cycle method (2^−ΔΔCt^) normalized to the housekeeping gene TFRC, since the TFRC genes were stable under different conditions.^[^ ^85^, ^86^ ^]^

### Numerical Simulations

The layout of the ITO electrode was modeled and optimized by using COMSOL (COMSOL Multiphysics 5.3a, COMSOL Multiphysics GmbH, Zürich, Switzerland). Fluid flow was modeled in MATLAB (The MathWorks Inc., Natick, USA). Additional information on the numerical simulations is reported in the Supporting Information.

### Statistical Analysis

All results are presented as mean values ± standard deviations (s.d.). A Mann–Whitney test was used to analyze the difference between two groups. Statistical analysis was performed using Prism GraphPad 9 (*p < 0.05 and **p < 0.01).

## Conflict of Interest

The authors declare no conflict of interest.

## Author Contributions

W.W., A.H., and M.M.M. conceived and designed the research. W.W. designed and developed the TEER sensor under the supervision of F.C. and M.M.M. W.W. designed and developed the microfluidic platform, performed experiments, and performed the data analysis under the supervision of M.M.M. All authors wrote the manuscript and approved the final manuscript.

## Supporting information

Supporting Information

Supplemental Video 1

Supplemental Video 2

## Data Availability

The data that support the findings of this study are available from the corresponding author upon reasonable request.
