# Supplementary material for: 3D In Vitro Blood‐Brain‐Barrier Model for Investigating Barrier Insults
Source: Adv Sci (Weinh). 2023 Feb 13;10(11):2205752. doi: 10.1002/advs.202205752 (PMC10104638; doi:10.1002/advs.202205752)
Supplement: Supplementary file 1 — Supporting Information [file ADVS-10-2205752-s003.pdf]

ADVANCED SCIENCE
Open Access

Supporting Information

for *Adv. Sci.*, DOI 10.1002/advs.202205752

3D In Vitro Blood-Brain-Barrier Model for Investigating Barrier Insults

*Wei Wei, Fernando Cardes, Andreas Hierlemann and Mario M. Modena**

Supporting Information

3D *in vitro* blood-brain-barrier model for investigating barrier insults

*Wei Wei, Fernando Cardes, Andreas Hierlemann and Mario M. Modena**

ETH Zürich, Department of Biosystems Science and Engineering, Bio Engineering Laboratory, Basel 4058, Switzerland
E-mail: mario.modena@bsse.ethz.ch

Supplementary Information

Fabrication of PDMS layers: To form the microchannel layer, the mixed and degassed PDMS (elastomer: curing agent in a 10:1 (w/w) ratio) was spin-coated on a glass slide, which had been previously coated with a sacrificial layer of 5% polyvinyl alcohol (Sigma-Aldrich, Buchs, Switzerland). The PDMS was spin-coated at 650 rpm for 45 s to obtain a 100-µm-thick film and subsequently cured in an oven at 80 °C for 2 h. The cured PDMS film was then cut by using a CO_2 laser cutter (Universal Laser Systems, Vienna, Austria) to pattern the geometry of the microchannel. As the PVA is water soluble, the PDMS film was peeled off from the glass slide after immersing the slide in deionized (DI) water to release the microchannel layer, and then dried in an oven at 60 °C for 10 min.

The PDMS interlayer was fabricated by pouring 10 g of mixed and degassed PDMS (elastomer: curing agent in a 10:1 (w/w) ratio) into 145 mm Petri dishes (Greiner Bio-One, Gallen, Switzerland). After curing for 2 h at 80 °C, the PDMS was removed from the dishes, and the brain compartment and inlet /outlet structures were marked with the laser cutter before they were manually cut using a biopsy puncher.

The hanging-drop layer and the open reservoirs were cast from a 3D-printed master mold (Protolabs, Feldkirchen, Germany) by using soft lithography. The mixed and degassed PDMS (elastomer: curing agent in a 10:1 (w/w) ratio) was poured onto the master mold. After curing for 2 h at 80 °C, the PDMS hanging-drop layer and the PDMS open reservoirs were peeled off the master mold and cut into individual chips. The hanging-drop structures were punched through with a 3 mm biopsy punch.

Electrode fabrication: The glass-ITO coverslip electrodes were fabricated by using photolithography and wet etching. Positive photoresist S1813 was spin-coated on an ITO-coated coverslip and patterned by using a mask aligner and UV exposure. After resist development, the patterned glass-ITO coverslip was immersed into the etching solution (HCl (36%): HNO_3 (67%): H_2O = 10: 1: 36 (v/v/v)) for 120 s at room temperature, followed by photoresist stripping via acetone. The glass-ITO coverslips with patterned ITO electrodes were rinsed in DI water and dried. To reduce the contact resistance between the ITO electrodes and the spring connectors, 270-nm-thick platinum was deposited on the ITO contact pads on the glass-ITO coverslip by using an ion-beam evaporator and patterned with a shadow mask.

The top 270-nm-thick platinum electrodes were deposited on a 6”, 150-um-thick PET film by using an ion-beam evaporator and patterned with a shadow mask (Figure S3). After Pt deposition, the PET film was diced into individual PET-Pt electrode slides (15 mm × 21 mm)

by using the laser cutter. Three 2 mm-diameter holes were cut by the laser cutter on the electrode slides to realize the openings for the microchannel reservoirs and for the hydrogel compartment.

Chip assembly: To bond the glass-ITO electrode layer and the microchannel layer, the glass-ITO substrates were exposed to oxygen plasma (2 min, 30 W using a PDC-002, Harrick Plasma, New York, USA), which was followed by APTES (Sigma-Aldrich) treatment in a vacuum chamber. After being rinsed with DI water, the APTES-treated glass-ITO substrates were bonded with the plasma-treated microchannel layer.

The PET porous membrane was sandwiched between the microchannel layer and the PDMS interlayer by using uncured PDMS as a glue (elastomer: curing agent in a 10:3 (w/w) ratio). The PDMS glue was spin-coated on a glass slide (50 mm × 75 mm) at 3000 rpm for 90 s. The plasma-bonded, glass-ITO-microchannel assembly and the PDMS interlayer were carefully placed on the spin-coated PDMS glue layer to transfer a thin layer of uncured PDMS on the layers to be bonded. Then, the PET membrane and the two PDMS-coated layers were aligned, degassed for 30 min in a vacuum chamber, and cured at 80 °C for 1 h.

The PET-Pt electrode layer was treated with oxygen plasma and then immersed into a 3% APTES solution at 80 °C for 30 min to modify the surface properties. The layer was then bonded between the lower assembly and the hanging-drop layer by using oxygen plasma. Finally, the medium reservoirs were plasma bonded to complete the chip assembly.

TEER Platform Assembly: Eight chips were placed between a custom-made printed circuit board (PCB) and a chip holder. The PCB was designed in Altium Designer 17.0 and ordered from PCBWay (Hangzhou, China). Electrical connections between the PCB and the microfluidic devices were obtained by contacting the electrode pads from above by using spring-loaded pins (0956-0-15-20-75-14-11-0, Mill-Max Mfg. Corp., Oyster Bay, USA). The PCB featured eight rectangular openings (16 mm × 26 mm) to allow for medium refreshing, permeability measurements and visual access to the chips without disassembling the platform.

The microfluidic device holder was custom-made in PMMA, which had been structured by laser cutting. The holder was placed in a well plate (Nunc™ OmniTray™, Thermofisher Scientific, Reinach, Switzerland) for simple handling and compatibility with lab automation equipment. The microfluidic device holder and the well plate featured eight openings that were aligned with the PCB openings for visual examination of the cells by using an inverted confocal microscope. The openings allowed the water immersion objective to contact the bottom coverslip for high-resolution imaging. Finally, the platform was covered with a laser-cut

polystyrene lid to prevent medium evaporation from the microfluidic devices and to allow for cable connections to the TEER electrodes.

Fluid dynamics: To estimate the fluid dynamics through the microfluidic devices, the CFD module of COMSOL was used. The fluid flow through the microfluidic device was set as a laminar flow, since the microfluidic channel featured a low Reynold's number.[1] The properties of water (at 37°C) were used to estimate those of the media in the experimental setup. The fluid was considered incompressible, and the dynamic viscosity μ was set to 0.7 mPa·s.[2] From simulations, we obtained a hydraulic channel resistance of

$$R_{hydro} = 5.53 \times 10^{10}\ kg/(m^4 \cdot s) \quad (3)$$

After calculating the channel resistance, we used this parameter to estimate the pressure drop across the channel during tilting and to calculate the resulting flow rate in MATLAB. The inlet flow rate in the COMSOL simulation was then set to the calculated flow rate, as shown in Figure S6, to estimate the shear stress on the endothelial cell layer.

Current density: To estimate the current density across the cellular barrier, the secondary Current Distribution (CD) module was used in COMSOL. The properties of water (at 37°C) were used to estimate those of the media in the experimental setup with an electrolyte conductivity of 1.6 S/m [3]. The electrical double layer capacitances of the WE and SE were set to 0.0337 F/m^2, and those of the CE and RE were set to 0.25 F/m^2.[4, 5] Frequency domain perturbation studies were used to simulate the current density at frequencies from 10 Hz to 2000 kHz. Additional simulation results of the current distribution in the barrier area are shown in Figure S3.

Fluid-flow modeling: The flow rate through the microchannel and the shear stress on the permeable barrier area were determined numerically using MATLAB. The custom-made MATLAB script considered the hydraulic resistance of the microchannel, which was estimated in COMSOL, and the shear stress across the permeable area of the porous membrane Figure S2, 6). The shear stress distribution in the barrier area was simulated by using COMSOL, using the flow and pressure values calculated in MATLAB as input parameters.

The cell culture medium is incompressible, and the medium flow in the microchannel is considered a laminar flow. The laminar flow is driven by the height difference (Δh) of the medium levels in the two side reservoirs, which generates hydrostatic pressure differences (ΔP) in the microchannel. The hydraulic resistance (R_{hydro}) of the microchannel has been calculated. The volumetric flow rate (Q) can be expressed as:

$$Q = \frac{\Delta P}{R_{hydro}} \quad 4$$

The microchannel hydraulic resistance (R_{hydro}) was calculated by using COMSOL Multiphysics:

$$R_{hydro} = 5.53 \times 10^{10}\ kg/(m^4 \cdot s) \quad 5$$

The hydrostatic pressure difference (ΔP) is controlled by the height difference (Δh) of the medium levels in the reservoirs:

$$\Delta P = \rho g \Delta h \quad 6$$

As shown in Figure S6, Δh is determined by the height difference (Δh_r) of the two reservoirs upon tilting and the difference in the medium height in the reservoirs ($h_1 - h_2$):

$$\Delta h = \Delta h_r + (h_1 - h_2) \quad 7$$

Δh_r is expressed as:

$$\Delta h = l \cdot \sin\theta \quad 8$$

Here, l is the distance between the medium reservoirs.

$h_1 - h_2$ is expressed as:

$$h_1 - h_2 = -\frac{2 \cdot \int_0^t Q\, dt}{A} \quad 9$$

Here, A is the cross-sectional area of the medium reservoir.

Therefore, the volumetric flow rate (Q) can be expressed by:

$$Q = \frac{\rho g (l \sin\theta - \frac{2\int_0^t Q dt}{A})}{R_{hydro}} \quad 10$$

The flow rate (Q) was calculated numerically using MATLAB.

Supplementary Figures

BBB platform

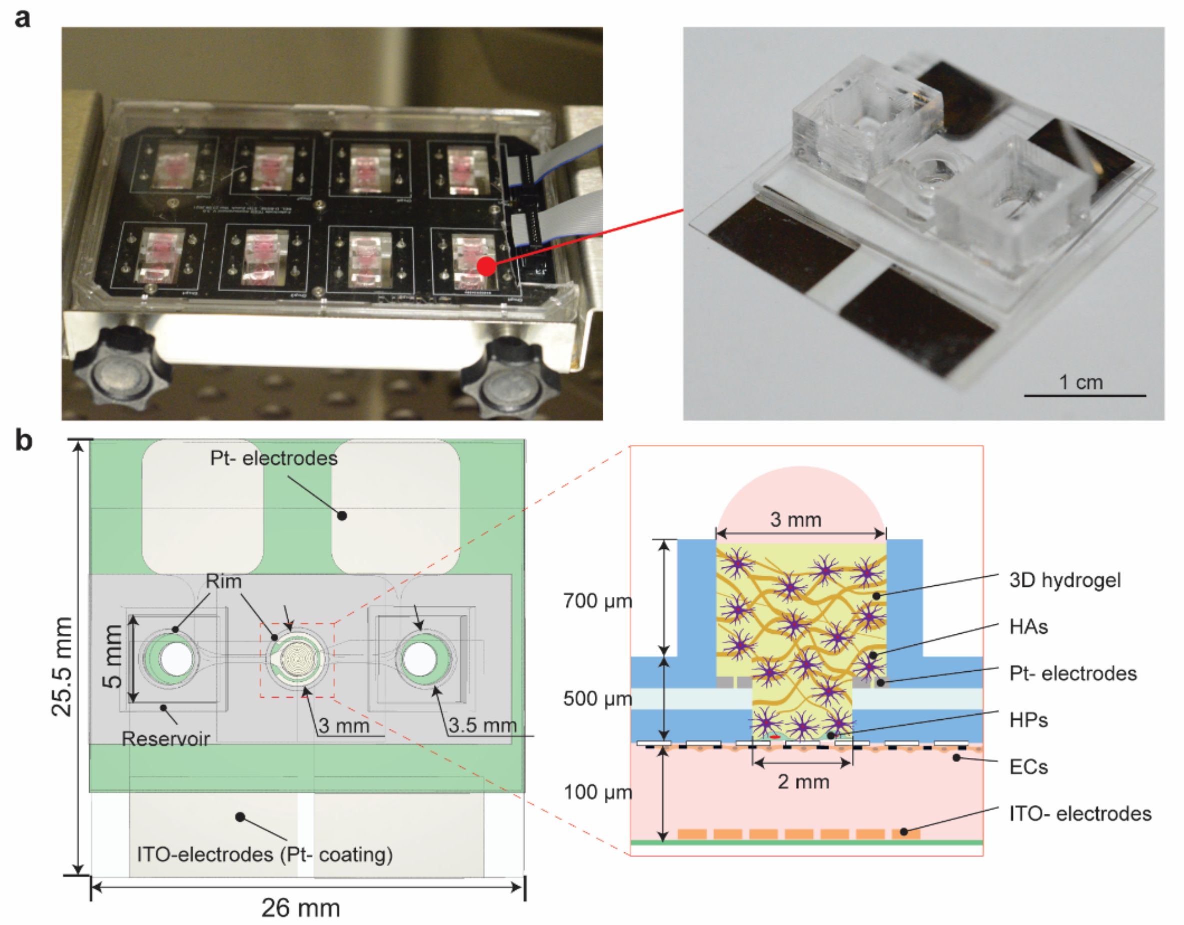

Figure S1. Design and dimensions of the BBB chip. a) Photograph of the BBB platform with 8 chips (left) and of a single chip (right). The BBB platform was composed of a top PCB to contact the on-chip electrode pads and a bottom layer to maintain the chips in position. Each chip featured four, Pt-coated electrode pads and large medium reservoirs; b) Design and dimensions of the BBB microfluidic chip (top view) and cell organization in the chip (side view). Cell-culture medium (in pink) was flown through the microfluidic channel and loaded on top of the brain compartment, which was filled with 3D hydrogel (yellow).

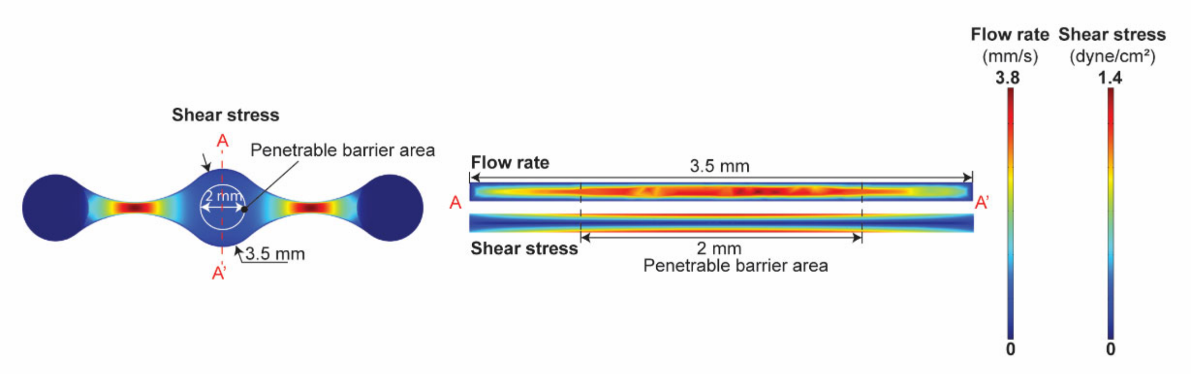

Figure S2. Simulation of shear stress using the COMSOL Multiphysics software. The diameter of the barrier area, which connected the vascular and brain compartments, was designed smaller than the total channel width so as to achieve uniform shear stress on the ECs forming the barrier.

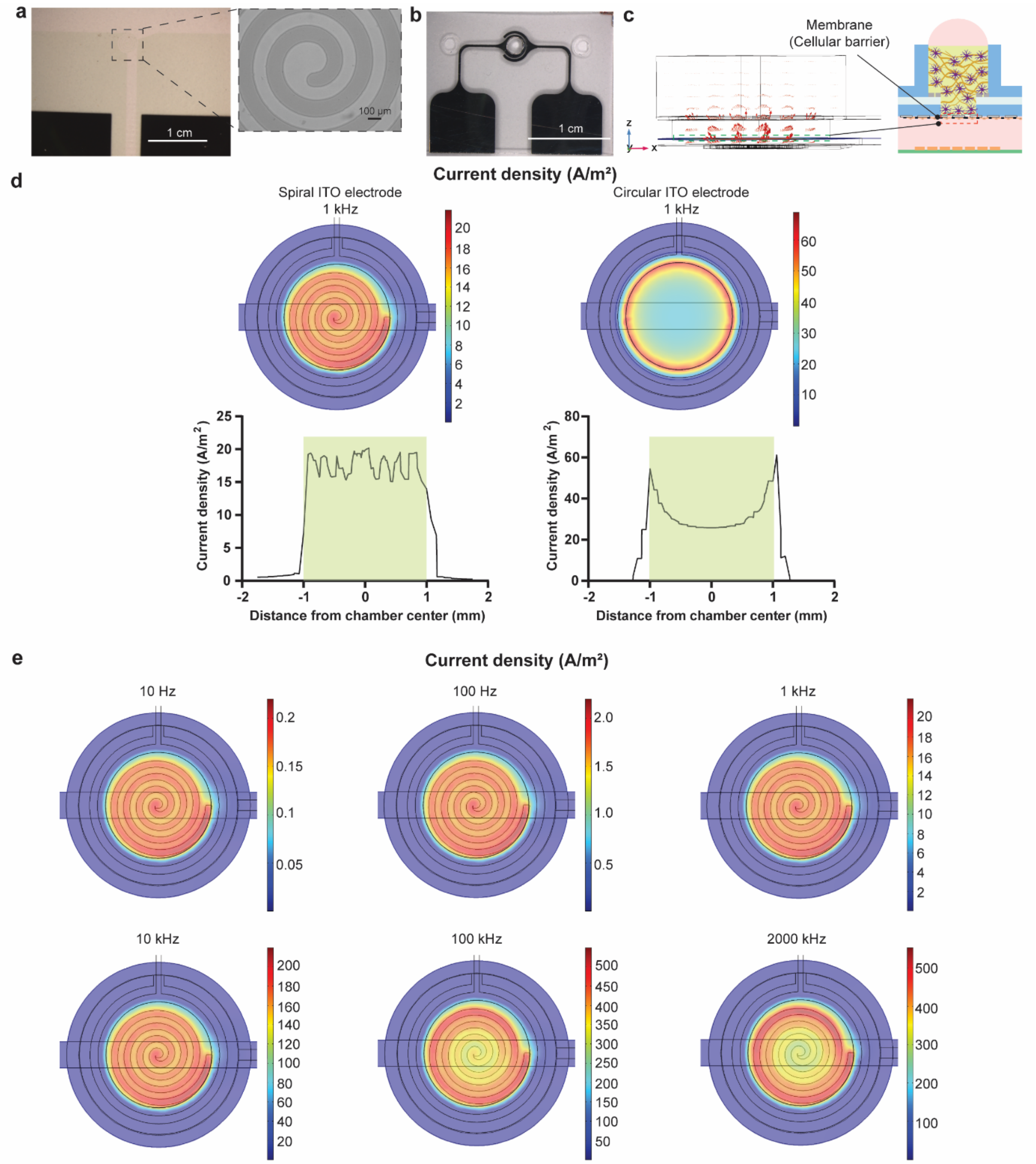

Figure S3. a) Photograph of the glass-ITO electrodes featuring large Pt contact pads. The electrodes were fabricated using transparent ITO on a 1.5# glass coverslip to enable high-resolution imaging. The Pt coating on the contact pads provided reliable electrical connections to external pins and reduced the overall system impedance. The working electrode in ITO featured spiral shape to obtain high uniformity current density across the barrier; b) Photograph of the PET-Pt top ring electrodes; c) FEM simulation of the current density in the chip. A sinusoidal voltage with different carrier frequencies was applied to the ITO working electrode. The red arrows indicate the current distribution from stimulation to sensing electrodes. The current passed through the membrane where the cellular barrier was formed and reported on the tightness of the barrier; d) Comparison of current density at the membrane layer using a spiral or circular working electrode along with the ring-based counter electrode. The spiral working electrode provided higher current uniformity across the cellular barrier at 1 kHz. The simulation was carried out by applying a 200-mV-amplitude sinusoidal stimulation. The plots indicate the root mean squared current density. The porous BBB area connecting the vessel and brain side is indicated through the shaded areas in the graph; e) FEM simulation of the current density across the membrane layer at different frequencies, obtained by applying a sinusoidal voltage stimulus of 200 mV amplitude. For signals below 100 kHz, the current density showed high uniformity across the porous membrane. However, for low frequencies (<1 kHz), the current densities were very low, which rendered signal detection difficult. All TEER values were calculated at 1 kHz. All FEM simulations were carried out using COMSOL Multiphysics.

Experimental setup

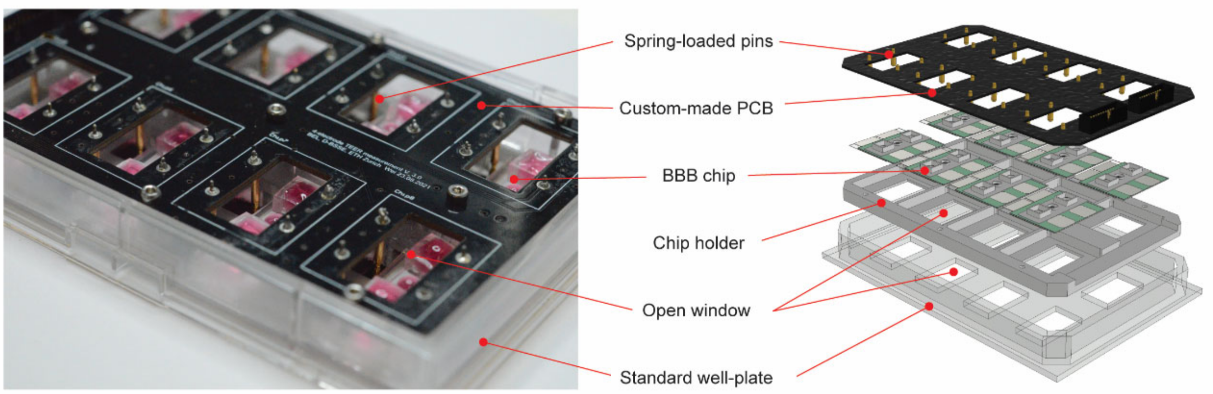

Figure S4. Assembly of the BBB platform. The platform is modular. 8 chips can be mounted between the connecting PCB and the chip holder. The spring-loaded pins on the PCB are aligned and pushed onto the electrode contact pads of the chips upon assembling the platform. The platform was arranged in a standard well-plate format for compatibility with laboratory automation and microscopy tools. Open windows in the PCB were aligned with the microfluidic-chip imaging areas so as to ensure continuous optical access. Open windows in the holder enabled the use of water immersion to allow for use of immersion objectives for high-resolution imaging.

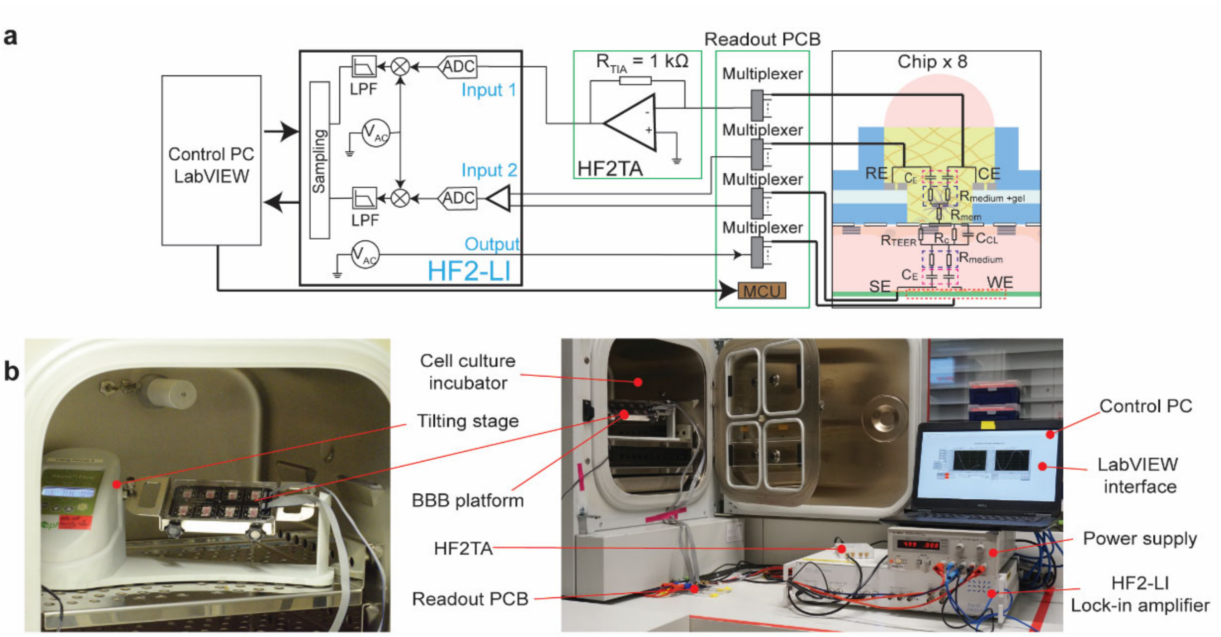

Figure S5. a) The electrical equivalent circuit of the experimental setup. A lock-in amplifier generates the AC stimulation signal, which is then routed to the chips by a custom-made PCB, where a microcontroller (MCU) is used to control the on-board multiplexers. The circuit is then completed by a HF2-LI transimpedance amplifier (HF2TA), which provides current-to-voltage (I-V) conversion. The HF2TA is then connected to the input of the lock-in amplifier to measure the current flowing in the system. The voltage drop across the barrier is measured using a set of dedicated electrodes on chip and by using a differential detection scheme. The input signals in the lock-in amplifier are digitalized (analog-to-digital converter, ADC), low-pass filtered (LPF), sampled, and subsequently recorded on a control PC; b) Photograph of the experimental setup, showing the BBB platform on the tilting stage in the incubator (left) and the TEER acquisition system (right).

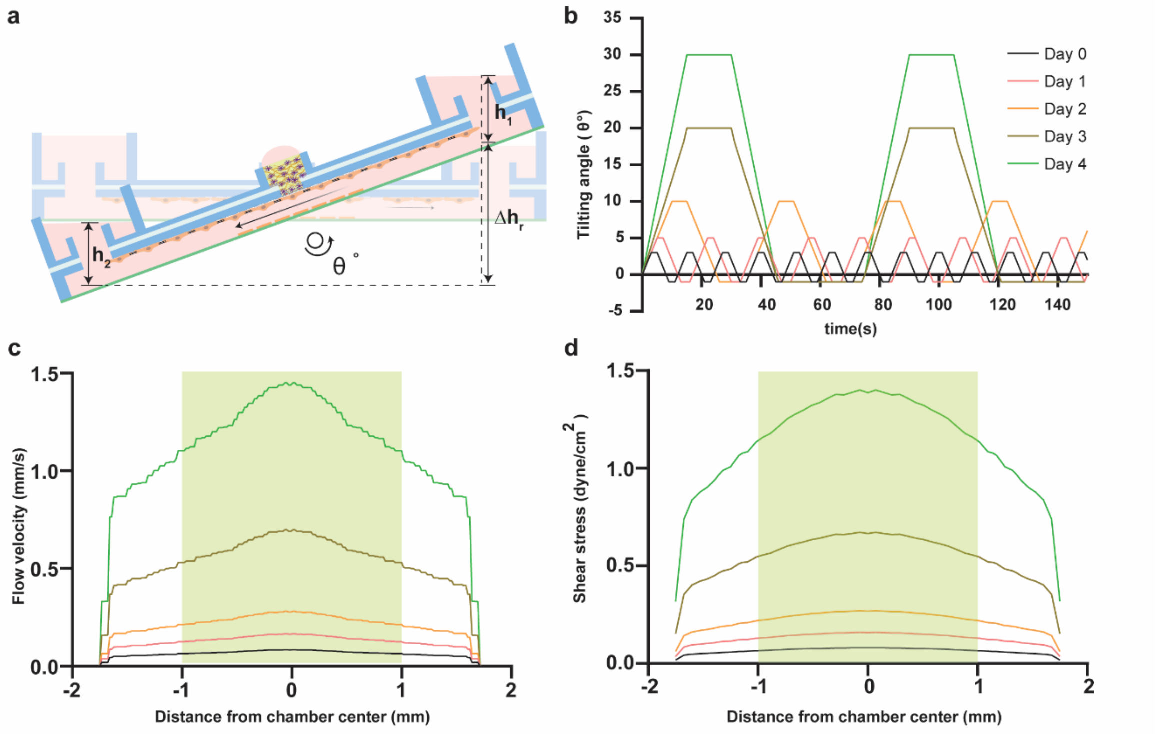

Figure S6. a) Tilting operation of the BBB platform for pump-free flow and dynamic culturing. Medium flow in the eight devices was generated by tilting the platform around an axis perpendicular to the microchannels; b) The tilting profile over time. To avoid damaging the EC layer during barrier formation, the flow rate was gradually increased every day by a stepwise increase of the tilting angles. An asymmetric tilting profile, with large positive tilting angles and small negative tilting angles, was selected to avoid equal-volume, bi-directional flow on the ECs; c) The flow rate at the location of the ECs in the middle of the barrier. The microchannel was wider than the barrier area to avoid having ECs exposed to extremely low flow rates; d) The corresponding shear stress on the ECs. The shaded areas in plots c) and d) indicate the porous surface connecting the vessel and the brain side of the BBB model.

3D Blood Brain Barrier

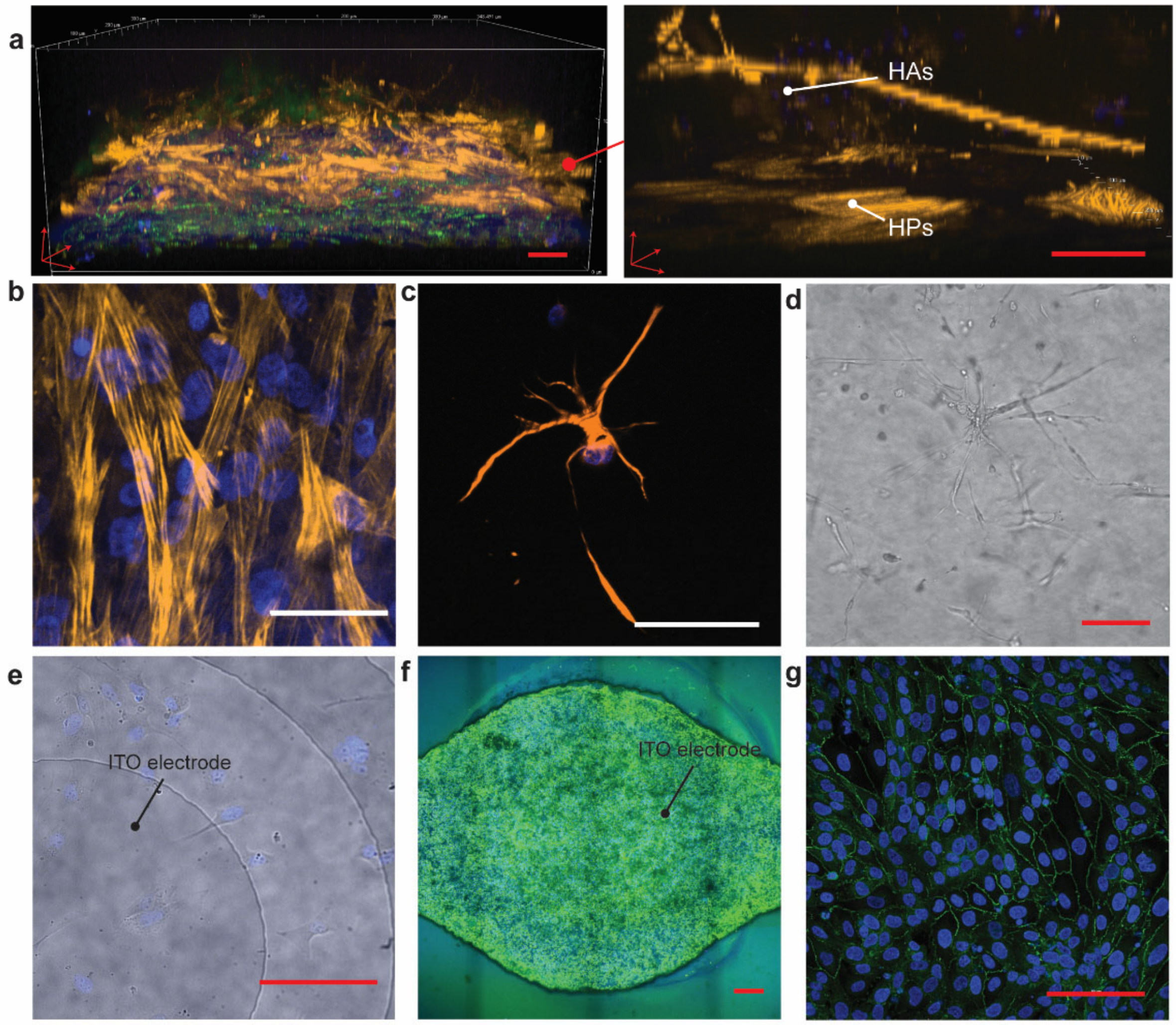

Figure S7. a) The multilayered 3D structure of the on-chip BBB. Endothelial cells formed a continuous monolayer at the bottom side of the porous membrane, and HPs grew on the opposite side of the porous membrane. HAs developed long branches, and the terminals of their processes reached the HPs; b) HPs grew adherent on the porous membrane; c, d) HAs in the hydrogel with GFAP and Hoechst staining (c) and a phase-contrast image (d) showing the characteristic star-like shape of HAs; e) After four days of EC culturing on chip and barrier formation, the electrodes were observed with wide-field microscopy to monitor EC growth (cell nuclei, Hoechst, in blue). The low number of ECs on the electrode confirmed that operating of the platform in hanging-drop mode during EC seeding prevented the attachment of large numbers of cells onto the electrodes; f-g) Immunofluorescence staining of the entire cellular barrier for tight junctions (ZO-1) and cell nuclei (Hoechst) in the microfluidic device, which shows that a continuous cell layer was formed across the entire BBB compartment. Scale bars: 100 µm (in red), 50 µm (in white).

TEER sensor characterization

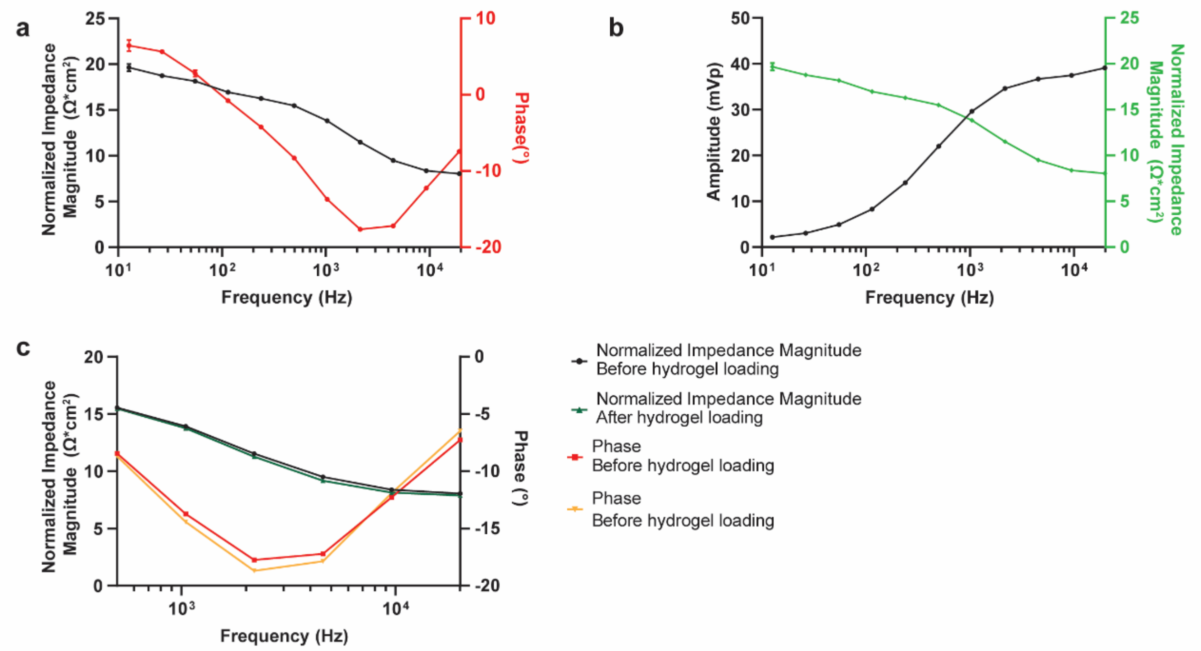

Figure S8. a) Normalized impedance and phase values of the signals acquired at different frequencies (ranging from 12.5 Hz to 20 kHz) while the platform was filled with PBS. The impedance was normalized by multiplying the impedance modulus with the active membrane area, as it is typically done for the calculation of the TEER values during barrier formation; b) The graph shows the voltage drop across the porous membrane and the corresponding normalized impedance as a function of frequency for a platform filled with PBS. The low signal amplitudes at <500 Hz prevented reliable measurements at low frequencies during barrier formation; c) Normalized impedance and phase values at different frequencies (500 Hz - 20 kHz) before (the platform was filled with HPM) and after hydrogel loading (the platform was filled with EAPM) into the brain compartment. The hydrogel in the compartment did not affect the sensor performance. Four measurements were acquired per time point.

TEER recording on chip

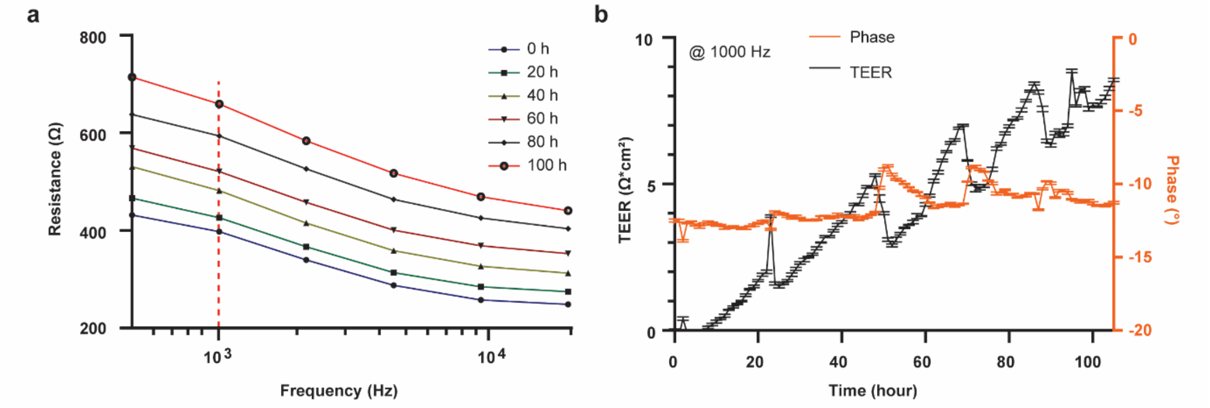

Figure S9. a) Example of impedance spectra, recorded during the BBB formation under dynamic coculture conditions. The impedance spectra were acquired in a range between 500 Hz and 20 kHz. 1 kHz impedance values were selected to calculate the TEER values, as shown in b; b) TEER and phase values were measured with the integrated sensor every hour under dynamic coculture conditions at 1 kHz. Four measurements were acquired per each time point. The graph shows mean values ± s.d..

TEER recording on one platform

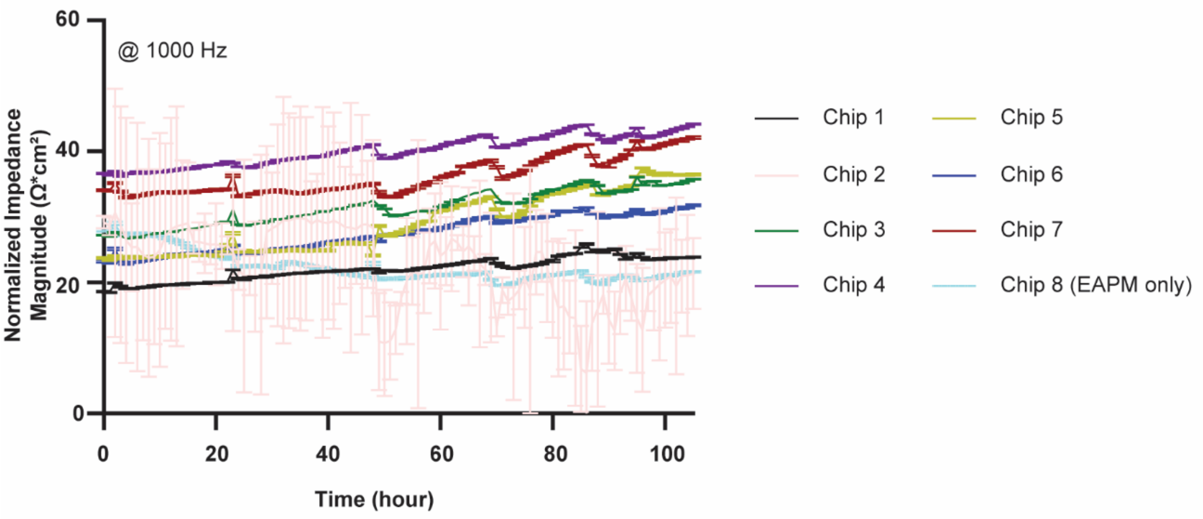

Figure S10. Normalized impedance of the signals from 8 chips in a platform, acquired by the integrated sensors. The impedance measurements were performed every hour at 1 kHz. Chips 1 to 7 were loaded with the triple co-culture, chip 8 was filled with EAPM as a control in the platform. Chips 1 and 3 to 7 showed an increase of the normalized impedance values, indicating the formation of the BBB. The impedance values of Chip 2 showed excessive fluctuations due to electrical connection issues. Four measurements were acquired per time point. The graph shows mean values ± s.d..

Standard curve of permeability measurements

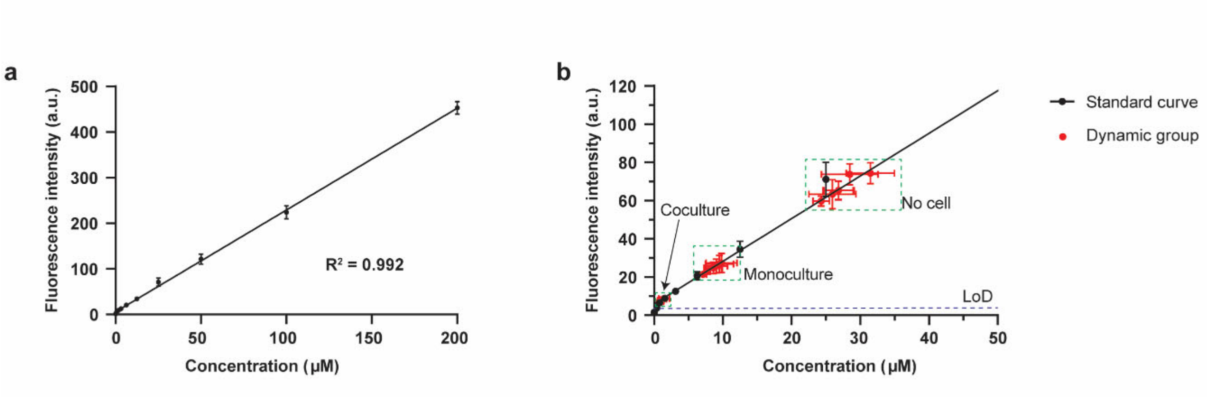

Figure S11. Standard curve of FITC fluorescence in the medium used for the permeability measurements. a) Standard curve of the FITC fluorescence in EAPM at different concentrations (0-200 μM, n = 6 for each concentration). The standard curve was acquired for each experiment and on the same measurement plate. b) Measurements of the fluorescence intensity of the collected samples for different cellular barrier conditions ("coculture", "monoculture" and "no cell", in red) under perfusion condition, and the points used to generate the standard curve (in black). The limit of detection (LOD) was calculated as 3 times the average of the background fluorescence signal (range: 1 - 2 a.u., n = 20).

Live, confocal imaging of the EC layer on chip under OGD conditions

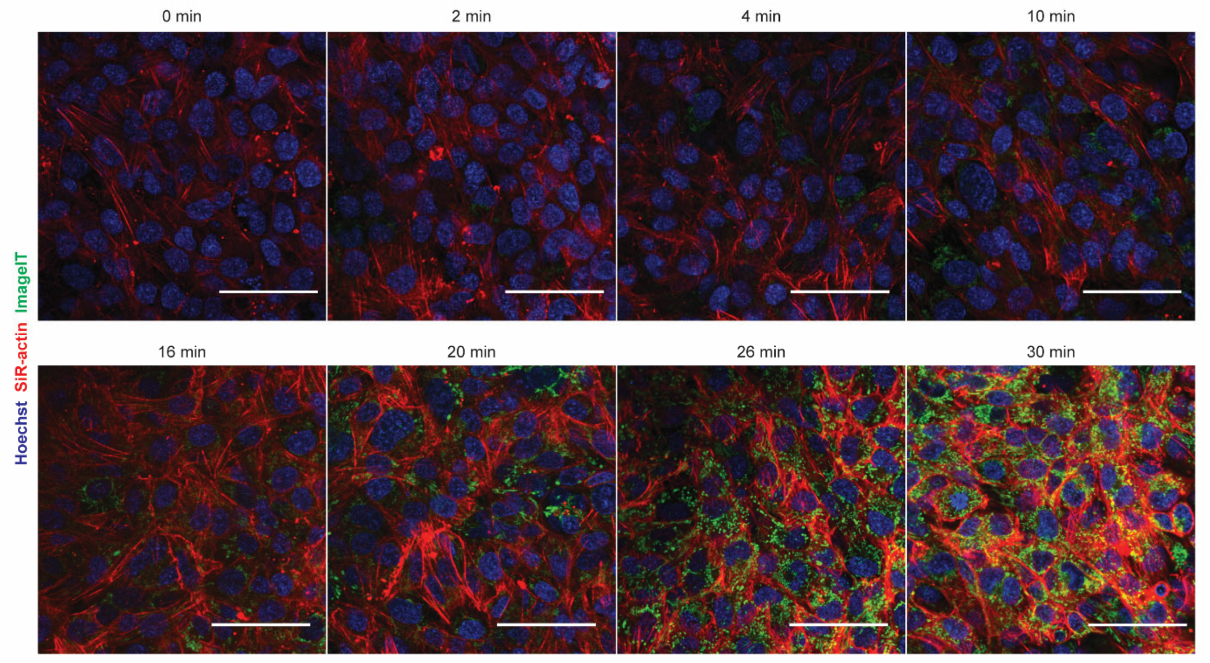

Figure S12. Live fluorescence images of the EC layer during OGD exposure. After 4 minutes of OGD exposure, the fluorescence signal of the hypoxia marker (ImageIT, green) appeared, which indicated that ECs had been under hypoxic stress. After that, the fluorescence signal of the hypoxia marker gradually increased, while F-actin (SiR-actin, red) began to be overexpressed, and actin filaments were rearranged. Strong actin signal, indicating the formation of stress fibers in the ECs, appeared after ~30 minutes of OGD exposure. In blue, cell nuclei (Hoechst). Scale bars: 50 µm.

Live, confocal imaging of Rhodamine 123 uptake by the endothelial cells during OGD exposure

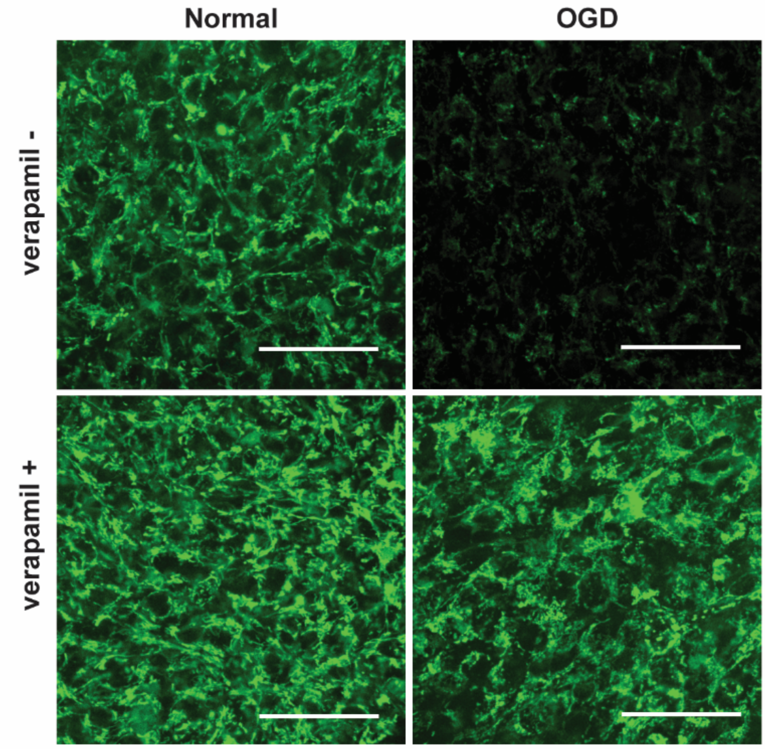

Figure S13. Fluorescence images showing Rhodamine 123 (green) uptake by the endothelial cells on chip under normal and OGD conditions. OGD exposure shows a much lower Rhodamine 123 uptake, which indicates an upregulation of P-gp activity. Upon exposure to the P-gp inhibitor Verapamil, an increase in the Rhodamine 123 signals could be detected, both under normal and OGD condition, which confirms P-gp activity. Scale bars: 50 µm.

Supplementary Tables

Primer sequence for RT-qPCR

Table S1. Primer sequences used for qPCR

Genes	Forward (5'-3')	Reverse (5'-3')	GenBank ID
ZO-1	TGCTGAGTCCTTTGGTGATG	AATTTGGATCTCCGGGAAGAC	NM_003257
VE-cadherin	AAACACCTCACTTCCCCATC	ACCTTGCCCACATATTCTCC	NM_001795
VEGFa	AGTCCAACATCACCATGCAG	TTCCCTTTCCTCGAACTGATTT	NM_001025366
TFRC	ACTTGCCCAGATGTTCTCAG	GTATCCCTCTAGCCATTCAGTG	NM_003234

Supplementary Video

Video S1. Live, confocal imaging of the EC layer on chip under OGD conditions for 1 h. The time-lapse video shows the EC layer during OGD exposure. In blue, cell nuclei (Hoechst); in red, F-actin (SiR-actin); in green, hypoxia marker (Image-iT Green Hypoxia Reagent). Scale bar: 50 µm.

Video S2. Live, confocal imaging of the EC layer on chip under OGD conditions for 3 h. In blue, cell nuclei (Hoechst); in red, F-actin (SiR-actin). Scale bar: 50 µm.

Data availability

The main data supporting the results in this study are available within the paper and its Supporting Information. The raw and analysed datasets generated during the study available for research purposes from the corresponding author on reasonable request.

Code availability

MATLAB was used to calculate the flow rate through the microchannel during the experiments. The codes used for the calculation can be provided on request.

References

[1] H. A. Stone, A. D. Stroock, A. Ajdari, *Annual Review of Fluid Mechanics* **2004**, *36*, 381.
[2] A. D. Wong, M. Ye, A. F. Levy, J. D. Rothstein, D. E. Bergles, P. C. Searson, *Front Neuroeng* **2013**, *6*, 7.
[3] G. Pucihar, T. Kotnik, M. Kandušer, D. Miklavčič, *Bioelectrochemistry* **2001**, *54*, 8.

[4] J. Martinez, A. Montalibet, E. McAdams, M. Faivre, R. Ferrigno, *2017 39th Annual International Conference of the IEEE Engineering in Medicine and Biology Society (EMBC)* **2017**, 3.
[5] B. Fan, B. Wolfrum, J. T. Robinson, *J Neural Eng* **2021**, *18*.
